# Application of *Safirinium N*-Hydroxysuccinimide Esters to Derivatization of Peptides for High-Resolution Mass Spectrometry, Tandem Mass Spectrometry, and Fluorescent Labeling of Bacterial Cells

**DOI:** 10.3390/ijms21249643

**Published:** 2020-12-17

**Authors:** Joanna Fedorowicz, Magdalena Wierzbicka, Marek Cebrat, Paulina Wiśniewska, Rafał Piątek, Beata Zalewska-Piątek, Zbigniew Szewczuk, Jarosław Sączewski

**Affiliations:** 1 Department of Chemical Technology of Drugs, Faculty of Pharmacy, Medical University of Gdańsk, Al. Gen. J. Hallera 107, 80-416 Gdańsk, Poland; 2 Faculty of Chemistry, University of Wrocław, ul. F. Joliot-Curie 14, 50-383 Wrocław, Poland; magdalena.wierzbicka@chem.uni.wroc.pl (M.W.); marek.cebrat@chem.uni.wroc.pl (M.C.); zbigniew.szewczuk@chem.uni.wroc.pl (Z.S.); 3 Department of Organic Chemistry, Faculty of Pharmacy, Medical University of Gdańsk, Al. Gen. J. Hallera 107, 80-416 Gdańsk, Poland; pauwis93@gumed.edu.pl (P.W.); js@gumed.edu.pl (J.S.); 4 Department of Molecular Biotechnology and Microbiology, Chemical Faculty, Gdańsk University of Technology, Narutowicza 11/12, 80-233 Gdańsk, Poland; rafpiate@pg.edu.pl (R.P.); beazalew@pg.edu.pl (B.Z.-P.)

**Keywords:** fluorescence microscopy, fluorescent probe, H/D exchange, live cell imaging, ionization tag, mass spectrometry, protein analysis, peptide labeling, tandem mass spectrometry

## Abstract

Mass spectrometry methods are commonly used in the identification of peptides and biomarkers. Due to a relatively low abundance of proteins in biological samples, there is a need for the development of novel derivatization methods that would improve MS detection limits. Hence, novel fluorescent *N*–hydroxysuccinimide esters of dihydro-[1,2,4]triazolo[4,3-*a*]pyridin-2-ium carboxylates (*Safirinium P* dyes) have been synthesized. The obtained compounds, which incorporate quaternary ammonium salt moieties, easily react with aliphatic amine groups of peptides, both in solution and on the solid support; thus, they can be applied for derivatization as ionization enhancers. *Safirinium* tagging experiments with ubiquitin hydrolysate revealed that the sequence coverage level was high (ca. 80%), and intensities of signals were enhanced up to 8-fold, which proves the applicability of the proposed tags in the bottom–up approach. The obtained results confirmed that the novel compounds enable the detection of trace amounts of peptides, and fixed positive charge within the tags results in high ionization efficiency. Moreover, *Safirinium* NHS esters have been utilized as imaging agents for fluorescent labeling and the microscopic visualization of living cells such as *E. coli* Top10 bacterial strain.

## 1. Introduction

The aim of proteomics is qualitative and quantitative analysis of proteins in complex samples. This field of study requires high-resolution separation methods and the selective identification of large organic molecules. Polyacrylamide gel electrophoresis (PAGE), high-performance liquid chromatography (HPLC), and mass spectrometry (MS) are the analytic techniques that are universally involved in proteomic studies. MS-based methods have been widely applied for the determination and quantification of a large number of proteins and peptides [1,2,3,4], for instance to identify new potential risks or prognostic biomarkers in medical diagnostics of diseases [5,6,7]. This approach allows the detection of protein modifications such as phosphorylation [8,9], oxidation [6], acetylation [10], and glycosylation [7]. Tandem mass spectrometry (MS/MS) is extensively employed in the analysis of peptides and proteins [11,12,13], often with the use of isotopic [14] as well as isobaric [9,15,16,17] tagging. More advanced techniques frequently utilized in proteomic studies involve isotopic dilution [5,18,19,20,21], hydrogen–deuterium exchange (HDX) [8,22] and multiple reaction monitoring (MRM) [7].

The MS determination of proteins in biological and clinical samples remains a challenging task due to the low abundance of peptides and matrix complexity. Sensitive MS analysis requires utilization of ionization tags (ITs), which increase ion signals and facilitate the determination of trace amounts of analytes [23,24]. Quaternary ammonium salts (QAS) are an example of ionization enhancers that can be used for this purpose [25,26,27]. Furthermore, these systems are useful in de novo peptide sequencing [28,29,30].

Aside from MS analytical methods, fluorescence sensing constitutes a valuable and important tool for analysis, control, and investigation, which is distinguished among the techniques dedicated to efficient, sensitive, and accurate detection of analytes in low concentrations. It gained widespread attention due to its cost-effectiveness, quick response time of fluorescence assays, operational simplicity, low limits of detection, wide linear range of analysis, and in situ monitoring ability. Fluorescence spectroscopy provides opportunities for fundamental research in numerous fields relevant to physical, chemical, biological, and medical sciences, such as clinical diagnostics, biotechnology, biochemistry, microbiology, environmental and analytical chemistry, materials science, as well as molecular and cell biology, comprising the real-time monitoring of cellular processes [31]. Owing to current hardware improvement in super-resolution microscopy, the lack of appropriate probes is considered as the main limitation of more advanced development. Thus, countless research groups have put a lot of effort into molecular design of novel small-molecule organic fluorophores [32,33,34,35] as well as fluorescent quantum dots [36,37] in order to increase the variety of routes to perform derivatization, tagging, and indirect probing in quantitative measurements. Fluorescent markers and labeled probes are widely employed as imaging agents for in vitro, in vivo, and ex vivo visualization of biological systems [38,39,40,41]. Considering these facts, there is a clear need for the rational design and preparation of new sensitive fluorescent probes that address challenges such as selective conjugatability and membrane permeability.

**Figure 1:**
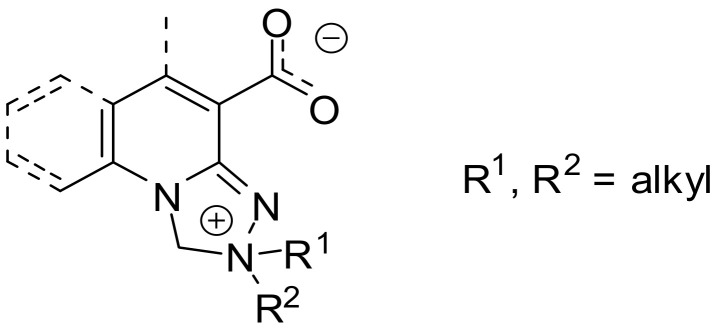
The general structure of *Safirinium* dyes (1,2,4-triazolo[4,3-*a*]pyridin-2-ium carboxylates).

Recently, we have reported the synthesis of fluorescent compounds based on 1,2,4-triazolo[4,3-*a*]pyridin-2-ium carboxylate system named *Safirinium* dyes (Figure 1) [42,43]. The novel derivatives demonstrate narrow and tunable absorption and emission bands, high-fluorescence quantum yields, relatively large Stokes shifts, and photostability. Moreover, they present advantageous features including extraordinary solubility in water, adjustable lipophilicity, simplicity in synthesis, and low cost of production, which make them useful for practical applications. Hence, this class of compounds may serve as fluorescent probes for the selective detection of secondary amines or formaldehyde, as well as reagents for labeling of lysine-containing peptides, *Bacillus subtilis* spores, and human skin structures. Moreover, *Safirinium* core upon conjugation with fluoroquinolone antibiotics or triterpenoic acids provide hybrid dual-acting antibacterial agents of improved bacterial cell penetration scheme [44,45,46] and cytotoxic agents targeting endoplasmic reticulum [47], respectively. The carboxyl group within the *Safirinium* scaffold can be readily converted into *N*-hydroxysuccinimide (NHS) ester form, which is reactive toward nucleophiles. Hence, NHS esters of *Safirinium* dyes in reactions with primary and secondary amines or thiols afford stable amide or thioester products, respectively. This approach has been successfully applied for the fluorescent labeling of proteinogenic amino acids [48]. Subsequent preliminary experiments indicated that the developed systems could be utilized as quaternary ammonium ionization tags, which would facilitate peptides identification due to the presence of permanent positive charge, i.e., quaternary nitrogen atom within triazolinium moiety [48].

Herein, we wish to combine both the permanent positive charge within the core structure and distinctive optical properties of the *Safirinium* dyes to design a compact, readily available ionization and fluorescent tags. The present paper is devoted to the development and application of novel probes suitable for mass spectrometry analyses as well as live-cell imaging.

## 2. Results and Discussion

### 2.1. Chemistry

**Scheme 1:**
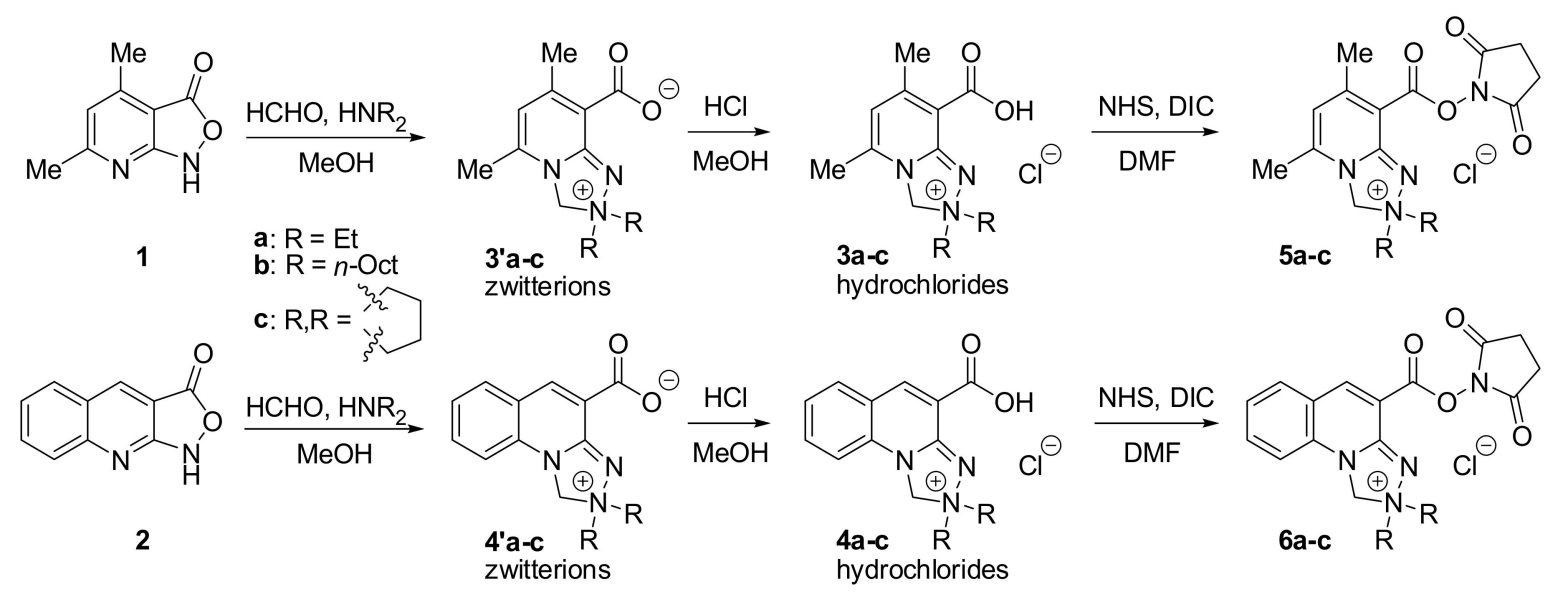
Synthesis of *Safirinium* dyes as well as their reactive *N*-hydroxysuccinimide esters.

A panel of *Safirinium* derivatives bearing diverse alkyl groups within the triazolium moiety (Scheme 1) have been synthesized according to our previously reported procedures [42,43]. Hence, the corresponding isoxazolones **1** and **2** were subjected to tandem Mannich–electrophilic amination reactions with formaldehyde and various secondary amines, such as diethylamine (**a**), dioctylamine (**b**), and pyrrolidine (**c**), to give *Safirinium P* (pyridine) and *Q* (quinoline) products **3′** and **4′**, respectively. Next, the synthesized products obtained in zwitterionic forms were converted into hydrochlorides **3** and **4** with use of methanolic HCl solution, and subsequently reacted with NHS and *N*,*N*’-diisopropylcarbodiimide (DIC) as a coupling agent to yield a series of active NHS *Safirinium* esters **5** and **6**.

**Scheme 2:**
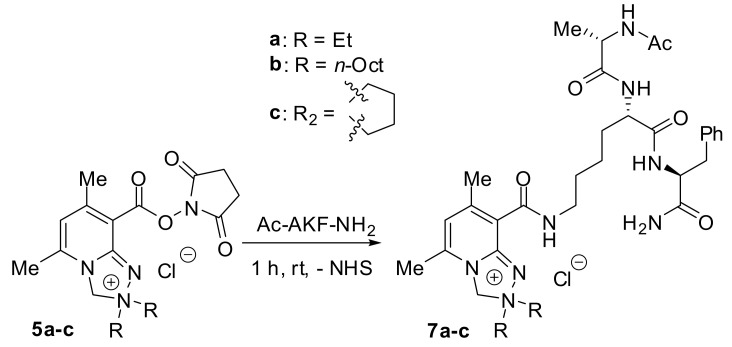
The reaction of active *Safirinium P N*-hydroxysuccinimide (NHS) esters **5a-c** with peptide Ac-AKF-NH_2_.

The model tripeptide, Ac-AKF-NH_2_, was tagged with *Safirinium P* esters **5a-c** in anhydrous dimethylformamide (DMF) at room temperature to form the corresponding **7a-c** derivatives (Scheme 2). The acetylated at the *N*-terminus peptide, which is also blocked at the *C*-terminus by conversion of the carboxyl group into an amide, reacts with NHS-activated agents, since it bears a free amine group in the lysine side chain. Since the tags with hydrophobic alkyl substituents increase the effectiveness of peptide ionization [24,28], in addition to the previously reported diethyl derivative 5a [42], we have synthesized compound **5b** that incorporates *n*-octyl substituents on the N2 nitrogen atom of the triazolinium ring. Additionally, novel pyrrolidine derivative **5c** was obtained for the reason that bicyclic spiro systems are resistant to Hoffman elimination, which significantly hinders mass spectra analysis for alkyl QAS under MS/MS conditions [49].

**Scheme 3:**
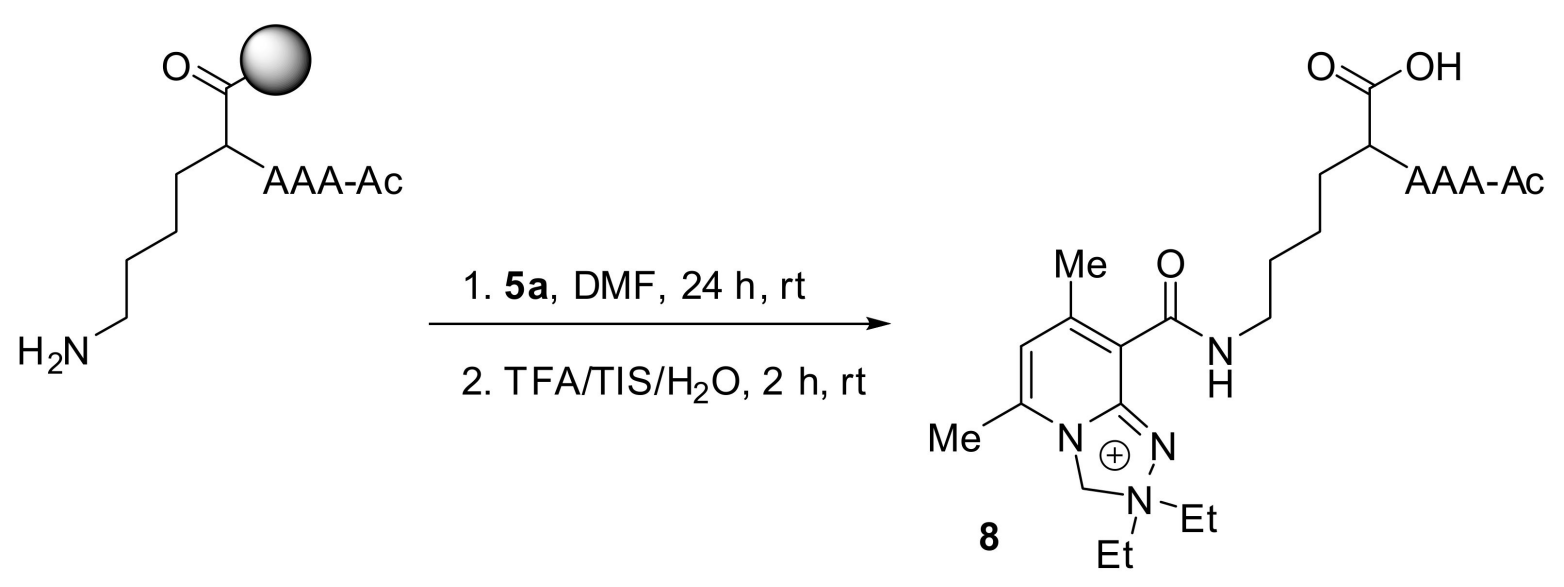
Tagging of Ac-AAAK-Wang with **5a** and its cleavage to yield compound **8**.

Tagging of a peptide immobilized on solid support was also completed. Hence, an exemplary tetrapeptide Ac-AAAK-Wang was labeled with reagent **5a** and subsequently cleaved from the resin (Scheme 3).

### 2.2. Mass Spectrometry Analysis

Electrospray ionization (ESI)-MS and ESI-MS/MS analyses of compound **5a**, AKF peptide conjugates **7a-c**, as well as AAAK peptide conjugate **8** have been performed.

#### 2.2.1. Analysis of Reactive NHS Ester **5a**

**Figure 2:**
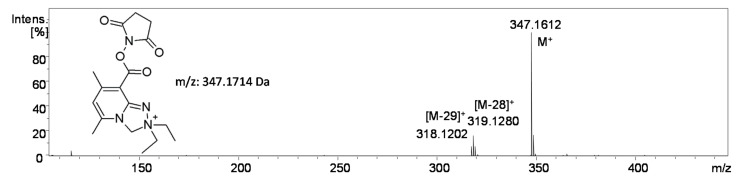
The electrospray ionization (ESI)-MS spectrum of compound **5a**.

**Figure 3:**
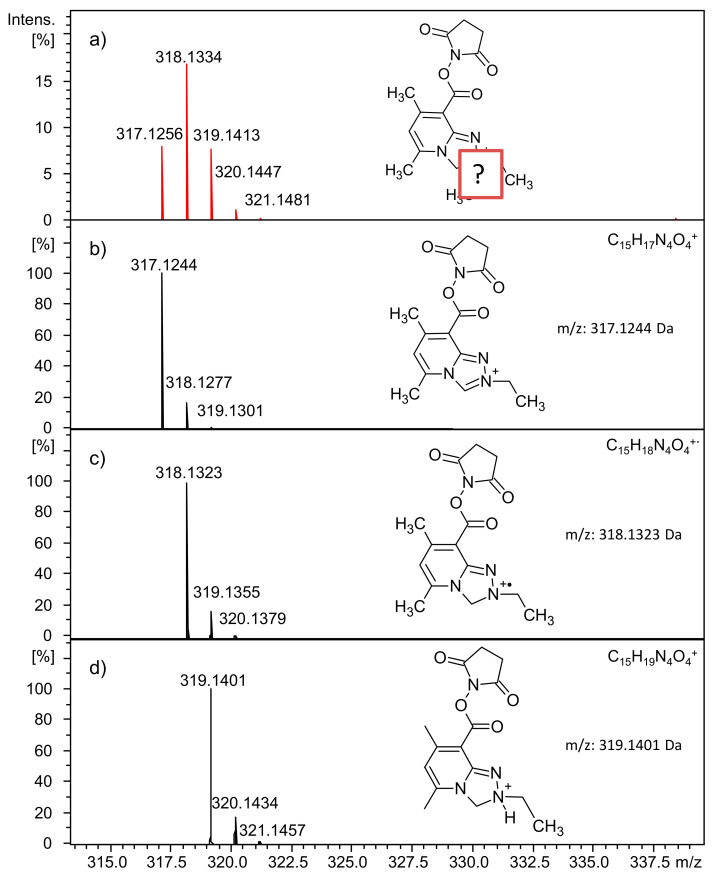
Enlarged fragment of the ESI-MS spectrum of **5a** (zoom (**a**)) with unknown structure of the fragmentation product (?) and the simulated isotopic patterns calculated for: (**b**) C_15_H_17_N_4_O_4_^+^, (**c**) C_15_H_18_N_4_O_4_^+**·**^, (**d**) C_15_H_19_N_4_O_4_^+^, that could correspond to this pattern.

ESI-MS spectrum of tag **5a** was measured on ESI-FT-ICR-MS instrument and is shown in Figure 2. We identified its [M]^+^ signal (*m*/*z* calc. 347.171, found 347.172); however, the spectrum also revealed an unusual peak distribution from 317 to 320 *m*/*z*. For their further identification, we have simulated the isotopic patterns (Figure 3) of C_15_H_19_N_4_O_4_^+^ (*m*/*z* = 319.140, panel d), which is the Hoffman elimination product of the analyzed compound, C_15_H_18_N_4_O_4_^+**·**^ (*m*/*z* = 318.132, panel c), the radical cation that may arise during the fragmentation, and C_15_H_17_N_4_O_4_^+^ (*m*/*z* = 317.124), which may be a product of charge remote fragmentation [50,51], leading to the alkene and H_2_ elimination (panel b). The supposed structures are given in Figure 3. The simulated isotopic patterns are in accordance with the observed pattern (panel a).

**Figure 4:**
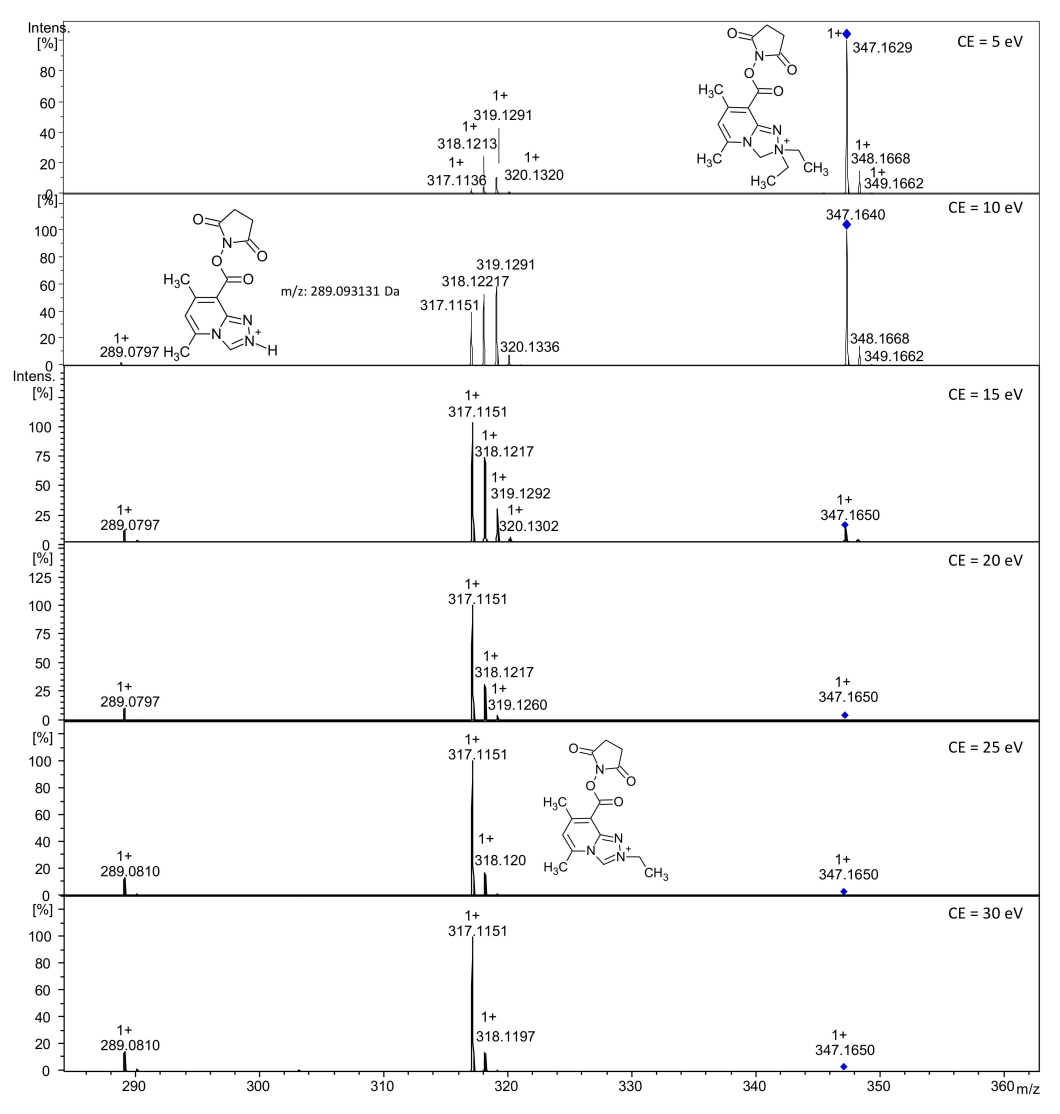
The ESI-MS/MS spectra of compound **5a** for a set of collision energies from 5 to 30 eV (collision-induced dissociation (CID) fragmentation, parent ion: 347.2).

In ESI-micrOTOF-Q MS/MS experiments with collision-induced dissociation (CID) fragmentation (parent ion 347.2) and collision energies from 5–30 eV (Figure 4) unusual peak distribution from 317 to 320 *m*/*z* is observed, which varies depending on applied collision energies (CE). For the collision energies of 5 eV and 10 eV, the highest daughter signal appears at 319.130, which is characteristic for the product of Hoffman elimination, i.e., elimination of CH_2_=CH_2_ from **5a** (Figure 3d). The second abundant fragmentation signal 318.122 corresponds to the radical cation (Figure 3c). With rising collision energy, these two signals disappear, and in 25 eV, a product of charge remote fragmentation (317.124 as in Figure 3b) is observed. The spectra reveal also signal 289.080, which corresponds to the product of the structure given in Figure 4 (*m*/*z* calc. 289.093).

**Figure 5:**
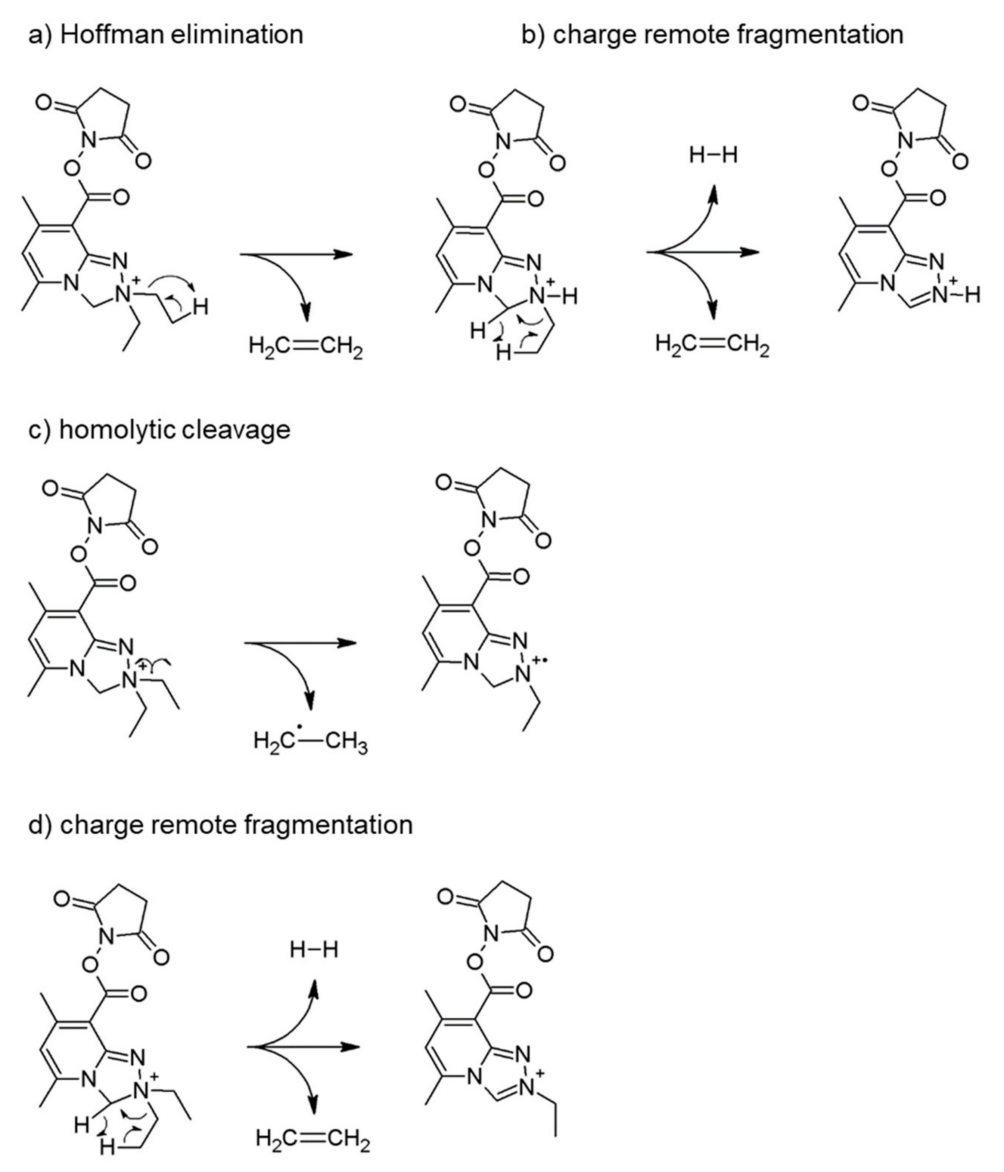
The fragmentation schemes of compound **5a** for a set collision energies from 5 to 30 eV (CID fragmentation, parent ion: 347.2).

The proposed mechanisms of fragmentation reactions, based on the observed patterns, are shown in Figure 5: scheme (a) illustrates the Hoffman elimination of ethylene from **5a**, while equation (b) presents the simultaneous elimination of second ethylene molecule and H_2_ during charge remote fragmentation. The homolytic cleavage that leads to the radical cation and ethylene radical formation is shown in scheme (c). Finally, equation (d) corresponds to the charge-remote fragmentation of compound **5a**, which gives rise to the formation of the most abundant product (*m*/*z* 317.124) for high-energy CID.

#### 2.2.2. Analysis of Conjugate **7a**, i.e., Peptide Ac-AKF-NH_2_ Tagged with Diethyl Derivative **5a**

**Figure 6:**
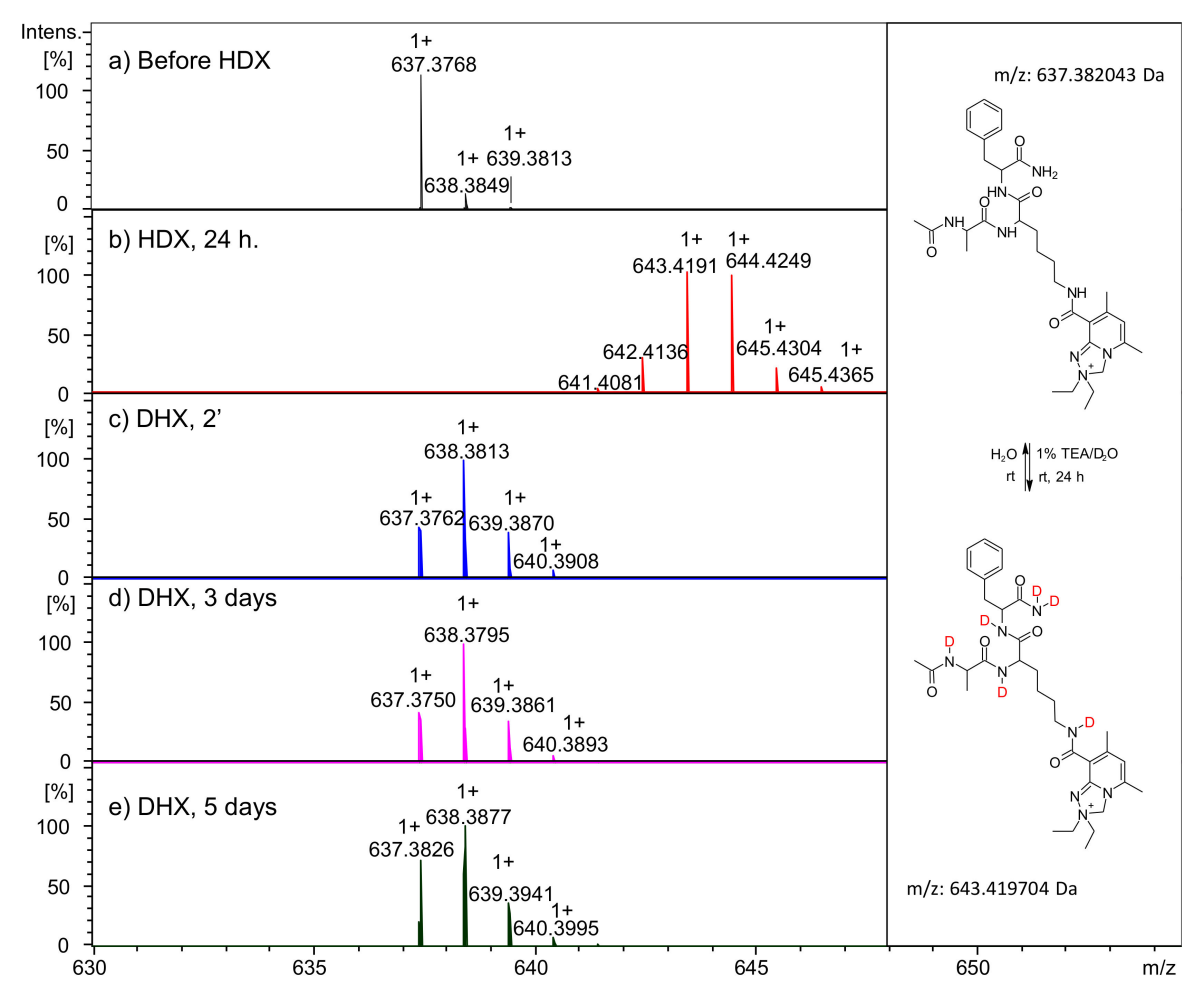
The isotopic patterns and reaction scheme of **7a**: (**a**) before hydrogen–deuterium exchange (HDX), (**b**) 24 h after HDX, (**c**) after re-dissolving in water, (**d**) 3 days after deuterium-hydrogen-exchange (DHX), and (**e**) 5 days after DHX.

Next, we have performed MS and MS/MS analyses of model peptide **7a** and its HDX subjected analogue to evaluate deuterated *Safirinium* system in applications that involve isotopic tagging. Hence, conjugate **7a** was incubated in 1% triethylamine (TEA)/D_2_O for 24 h at room temperature. Subsequently, after lyophilization, the sample was dissolved in water to examine the back-exchange process. In Figure 6, the following MS spectra are shown: (a) prior to HDX, (b) 24 h after the HDX procedure, (c) after re-dissolving in water, (d) 3 days after deuterium-hydrogen-exchange (DHX), and (e) 5 days after DHX. The reaction scheme is shown accordingly. Subsequently to HDX, the signal shifts 6 Da, which indicates that in the peptide conjugate **7a**, six hydrogen atoms have been exchanged to deuterons. After deuterium–hydrogen exchange, isotopic signals shift slowly to the previous values, which indicates that the deuterons are labile and not resistant to back-exchange in neutral pH. The DHX process takes much more time than HDX, and after 5 days of incubation in water solution, less than half of the deuterons are fully exchanged to hydrogens. Although it was not possible to obtain a stable isotopologue using HDX, the synthesis of this type of isotopic tagging reagent may be feasible. Considering the fact that the formation of 5-membered triazolinium ring by means of the tandem Mannich–electromphilic amination reaction requires formaldehyde, an application of deuterated formaldehyde would result in the development of a not expensive isotopic tag.

**Figure 7:**
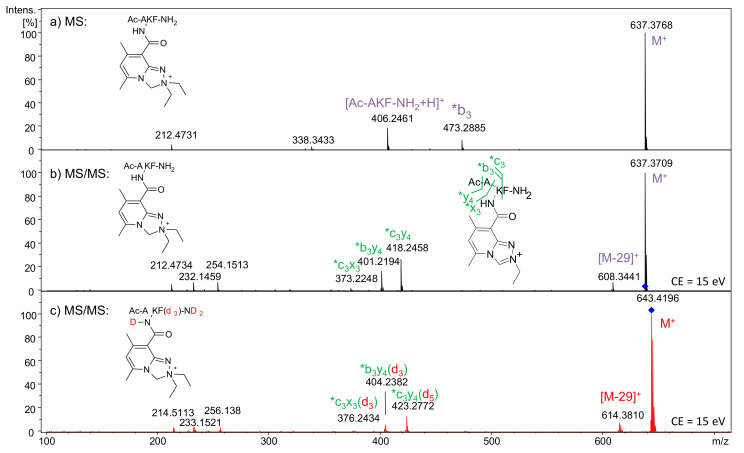
Mass spectrometry analysis of **7a**: (**a**) MS spectrum, (**b**) MS/MS spectrum with structures of daughter ions (parent ion—637.4, CE = 15 eV), (**c**) MS/MS spectrum of deuterated analogue (parent ion—643.2, CE = 15 eV).

The full MS spectrum of **7a** is shown in panel a of Figure 7. The most abundant signal 637.377 corresponds to the analyzed compound (*m*/*z* calc. 637.382). The signal at 406.246 refers to the untagged peptide Ac-AKF-NH_2_ ([M+H]^+^ calc. 406.245), while 473.289 indicates the *b_3_ ion of the studied peptide conjugate.

Panel b represents the MS/MS spectrum of the parent ion—637.4, CE = 15 eV. Hence, CID fragmentation reveals peaks representing the loss of 28.29 and 30 Da (observed signals 607–609 *m*/*z*, marked in the spectrum as [M-29]^+^), which correspond to the Hoffman elimination, homolytic cleavage, and charge remote fragmentation, respectively, as in the case of **5a**. Analogously, we have observed the change of the peak pattern with increasing collision energy. Furthermore, we identified the *b_3_y_3_, *c_3_y_3_, and *b_3_x_3_ ions, whose structures are schematically given in Figure 7b).

We have also performed an MS/MS experiment for the deuterated analogue: parent ion—643.2, CE = 15 eV (Figure 7c). Similarly, the same fragmentation pattern as for the non-deuterated analogue was observed, which confirms that the exchangeable hydrogens are not located on ethyl groups of *Safirinium P* system. In the MS/MS spectrum, there were also present signals that correspond to the deuterated analogues of daughter ions, as indicated previously in the spectrum presented in panel (b). Hence, shifts of 5 Da for the *c_3_y_3_-(d_5_) and 3 Da for the *b_3_y_3_-(d_3_) and *b_3_x_3_-(d_3_) ions, with respect to the number of labile protons in the daughter deuterated ions, were observed in the spectrum, respectively.

#### 2.2.3. Analysis of Conjugate **7b**, i.e., Peptide Ac-AKF-NH_2_ Tagged with Dioctyl Derivative **5b**

**Figure 8:**
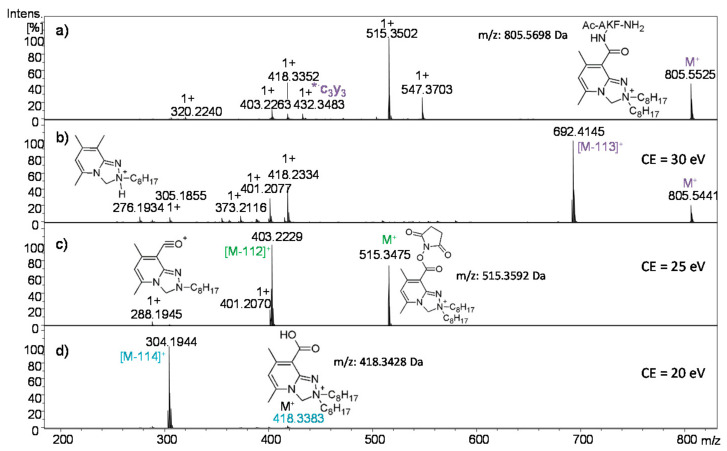
Mass spectrometry analysis of **7b**: (**a**) MS spectrum, (**b**) MS/MS (parent ion—805.5, CE = 30 eV), (**c**) MS/MS **5a** (parent ion—515.3, CE = 25 eV), and (**d**) MS/MS of **3a** (parent ion—418.3, CE = 20 eV).

MS and MS/MS analyses of the **7b** conjugate are shown in Figure 8. The panel (a) represents the mass spectrum of unreacted compound **5b** and the tagged peptide **7b**. The signal at *m*/*z* of 805.552 corresponds to the tagged peptide (*m*/*z* calc. 805.569), while the signals at *m*/*z* 515.350 and 418.335 indicate the unreacted **5b** ester (*m*/*z* calc. 515.359) and compound **3b** formed by hydrolysis of **5c**, respectively. We have also identified the *^·^c_3_y_3_ radical ion, which is the product of the fragmentation of **7b**, i.e., the loss of one octyl group.

Panel b represents the MS/MS spectrum of **7b**: parent ion: 805.5, CE = 30 eV. The CID fragmentation of **7b** results in the peak pattern (signals 691–693 *m*/*z*), which corresponds to the loss of 113, 114, and 115 Da. As in the cases of compounds **7a** and **5a**, the observed pattern results from Hoffman elimination, homolytic cleavage, and charge remote fragmentation, respectively. The change of peak pattern with respect to increasing collision energy, from 20 to 30 eV, has been analyzed. Similarly to the previous experiments, the most abundant peaks shifted to lower *m*/*z* values; however, according to the MS/MS spectrum of **7b**, other fragmentation products occur in both MS and MS/MS spectra. For example, the structure of one of the low mass fragmentation products (276.193) is shown in Figure 8b.

The MS/MS spectrum of the unreacted probe **5b** obtained with a collision energy of 25 eV shows signals at 515.3 and 288.195, which correspond to the parent and daughter ions as depicted in Figure 8c. The characteristic *Safirinium P* system fragmentation pattern leading to the ions with *m*/*z* from 401 to 403, as well as shift to lower *m*/*z* with respect to rising collision energy, have been observed. However, it should be pointed out that lower CE are needed for the fragmentation of **5b** compared to the conjugate **7b**.

Finally, an MS/MS experiment of ion *m*/*z* = 418.336, which corresponds to compound **3a**, has been performed. The spectrum at collision energy of 20 eV is shown in Figure 8d. In case of this spectrum, the characteristic fragmentation leading to the formation of ions with *m*/*z* from 304 to 306 has been evidenced. Similarly to other *Safirinium P* systems presented above, a shift to lower *m*/*z* values with rising collision energy has been recognized.

Summing up, a **5b** ionization tag provides less information regarding the analyzed peptide than probe **5a**, since the diethyl tag gives a single daughter ion, and its fragmentation requires higher CE when compared to the dioctyl counterpart.

#### 2.2.4. Analysis of Conjugate **7c**, i.e., Peptide Ac-AKF-NH_2_ Tagged with Pyrrolidine Derivative **5c**

**Figure 9:**
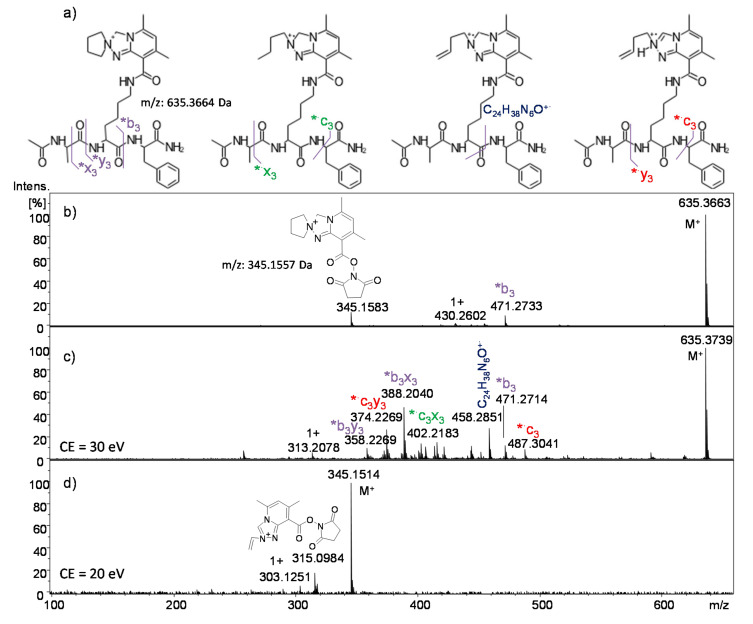
Mass spectrometry analysis of **7c**: (**a**) the postulated daughter ion structures (**b**) MS spectrum, (**c**) MS/MS (parent ion: 635.4, CE = 30 eV), (**d**) MS/MS of **5c** (parent ion: 345.1, CE = 20 eV.

Finally, a cyclic *Safirinium P* analogue **7c** has been examined. This conjugate was expected to give different MS/MS results than the *Safirinium P* analogues described above, since it incorporates an azoniaspiro system. As indicated earlier by Setner et al. [52], the azoniaspiro systems do not undergo the Hoffman elimination. The MS spectrum of crude compound **7c** is presented in Figure 9b. The most abundant signal in the spectrum at *m*/*z* 635.366 corresponds to the parent ion **7c** (*m*/*z* calc. 635.367), while the low abundant signal at *m*/*z* = 471.273 indicates its daughter ion *b_3_. There is also a signal at *m*/*z* 345.158, which corresponds to the unreacted probe **5c**. Subsequent MS/MS spectrum of the peptide conjugate was measured for the parent ion 635.4 (CE = 30 eV), as shown in Figure 9c. In the case of azoniaspiro *Safirinium P* analogue **7c**, neither Hoffman elimination nor charge remote fragmentation, which are characteristic for other *Safirinium* compounds, has been observed. Along with the daughter ion *b, observed also in the MS spectrum, the MS/MS experiment was revealed *b_3_y_3_ and *b_3_x_3_ ions. Additionally, spectrum analysis proved fragmentation mechanisms that lead to homolytic cleavage in the azoniaspiro system with the formation of daughter radical ions *^·^c_3_y_3_, *^·^c_3_x_3_, and the ion with a given molecular formula: C_24_H_38_N_6_O^+·^. The structures of the daughter ions are shown in panel (a). It is probable that the homolytic cleavage occurs during CID fragmentation of azoniaspiro *Safirinium P* conjugates, which results in electron donation from *Safirinium P* system to the peptide chain. Hence, further fragmentations lead to c and x type ions, similarly to electron transfer dissociation processes described in the literature [53].

**Figure 10:**
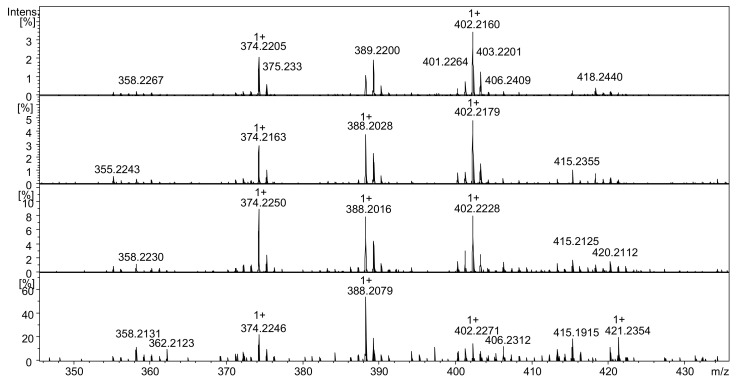
Fragmentation patterns in ESI-MS/MS spectra of **7c** (parent ion: 635.4) at collision energies from 20 to 30 eV.

Similarly to other *Safirinium P* derivatives and peptide conjugates described above, we analyzed how peak patterns change with rising collision energies (Figure 10). Hence, CE were set from 20 to 30 eV, since effective fragmentations required higher collision energies. In general, more sophisticated fragmentation patterns have been observed compared to previously analyzed *Safirinium P* conjugates, which implies more complicated fragmentation mechanism. However, CE increase did not affect the characteristic signal pattern as much as in previous analyses.

Finally, MS/MS analysis of the parent ion for ester **5c** (345.1, CE = 20 eV) was performed. When compared to the remaining *Safirinium P* NHS esters, the characteristic fragmentations were not observed, and analysis required higher CE. At 20 eV, the ester starts to fragment, giving the daughter ion at *m*/*z* 315.098. Its probable structure is shown in the spectrum (Figure 9d).

In comparison to peptide conjugates **7a** and **7b**, unwanted Hoffman elimination and charge remote fragmentation do not occur in analyses of **7c**; however, homolytic cleavage leads to rather complicated spectra. Similarly to an analysis of conjugate **7a**, in the spectra of compound **7c***,* only signals of daughter ions containing the tagged lysine residue were enhanced, while the remaining ions containing alanine or phenylalanine residues were not visible; hence, peptide sequencing would not be possible. However, the *Safirinium P* system creates characteristic c and x type ions that usually are common for electron transfer dissociations (ETD).

#### 2.2.5. Analysis of Conjugate **8**, i.e., Tetrapeptide Ac-AAAK Tagged with Diethyl Derivative **5a**

**Figure 11:**
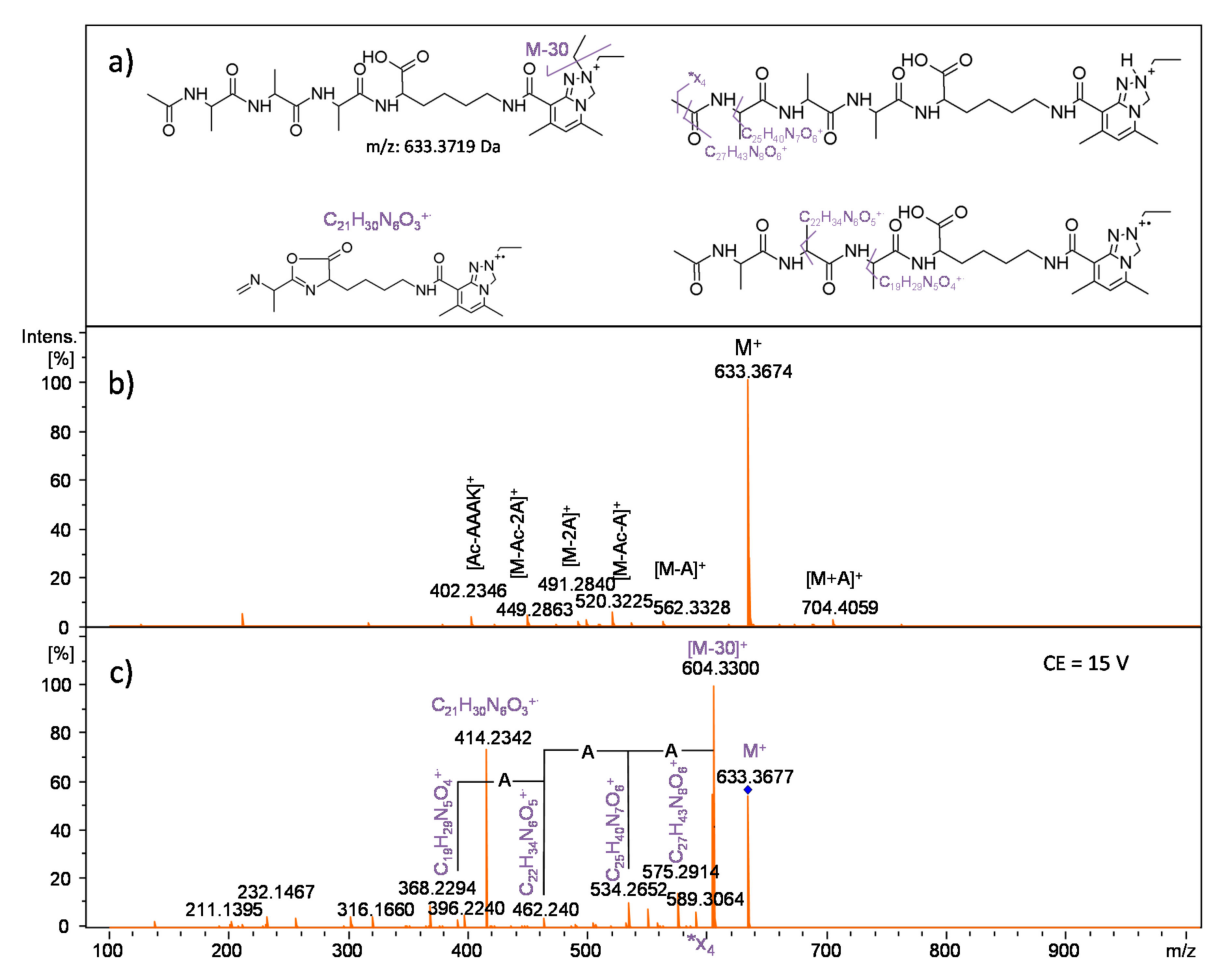
Mass spectrometry analysis of **8**: (**a**) general structure and fragmentation pattern, (**b**) MS, (**c**) MS/MS (CE = 15 V).

Electrospray Fourier-Transform Ion-Cyclotrone-Resonance MS (ESI-FT-ICR-MS) and MS/MS analyses of product **8** are presented in Figure 11. Panel (a) includes structures that illustrate fragmentation pattern. Panel (b) shows MS spectrum of **8**, i.e., the molecular ion (*m*/*z* calc. 633.3719, found 633.3674). The spectrum presents also signals that correspond to synthesis co-products, including acetylated and non-acetylated peptide intermediates with deletions of alanines or even with its additional insertion (704.406).

The MS/MS spectrum of the parent ion 633.4 obtained with collision energy of 15 eV is shown in panel (c). The signal pattern from 603 to 605 Da indicates losses of 30, 29, and 28 Da, respectively. The same fragmentation patterns, i.e., Hoffman elimination, homolytic cleavage, and charge remote fragmentation were observed for probe **5a** and peptide conjugate **7a**. Similarly to other *Safirinium P* derivatives, a rise in collision energy resulted in abundance change, indicating that the main fragmentation product moves from homolytic cleavage product (*m*/*z* 604.333) to charge remote fragmentation product (*m*/*z* 603.325).

The MS/MS spectrum (Figure 11c) reveals an *x_4_ ion and peak that resulted from methyl group loss, i.e., ion C_27_H_43_N_8_O_6_^+^ (*m*/*z* 575.286), whose structure is shown in panel (a). Furthermore, non-typical series of daughter ions with neutral losses of subsequent alanine residues were observed, which are analogues of *z ions and *^·^z radical ions without side chains, i.e., alanine methyl groups. Finally, the spectrum presents a highly abundant signal at *m*/*z* 414.230, which based on mass spectrum simulations corresponds to a radical ion with the chemical formula: C_21_H_30_N_6_O_3_^+·^.

Similarly, to other peptide–*Safirnium P* conjugated systems, the label enhances signal intensities; however, under MS/MS conditions, also radical reactions and additional fragmentations occur, which results in atypical fragmentation patterns. In the case of the peptide conjugate **8**, peptide sequencing based on neutral losses was sophisticated albeit possible.

### 2.3. Derivatization of Ubiquitin Hydrolysate with Reagent **5a**

**Scheme 4:**
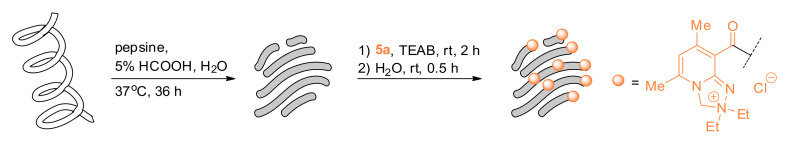
Ubiquitin hydrolysis and its ionization tagging with **5a**.

The ubiquitin pepsin hydrolysate was tagged with diethyl derivative **5a**. The reaction scheme along with ubiquitin hydrolysis procedure is shown in Scheme 4.

**Figure 12:**
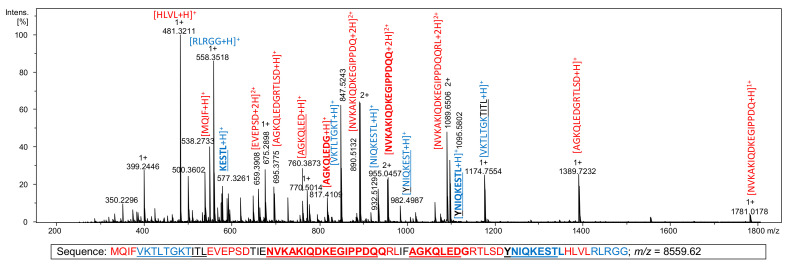
MS analysis of ubiquitin hydrolysate, upper panel: MS spectrum with peptide signals assigned; lower panel: ubiquitin sequence with hydrolysis sites marked.

Ubiquitin hydrolysate and its analogue tagged by **5a** have been analyzed by MS and LC-MS. The mass spectrum of ubiquitin hydrolysate is shown in Figure 12. The most abundant signals correspond to the products identified previously by Jaremko et al. [54].

**Figure 13:**
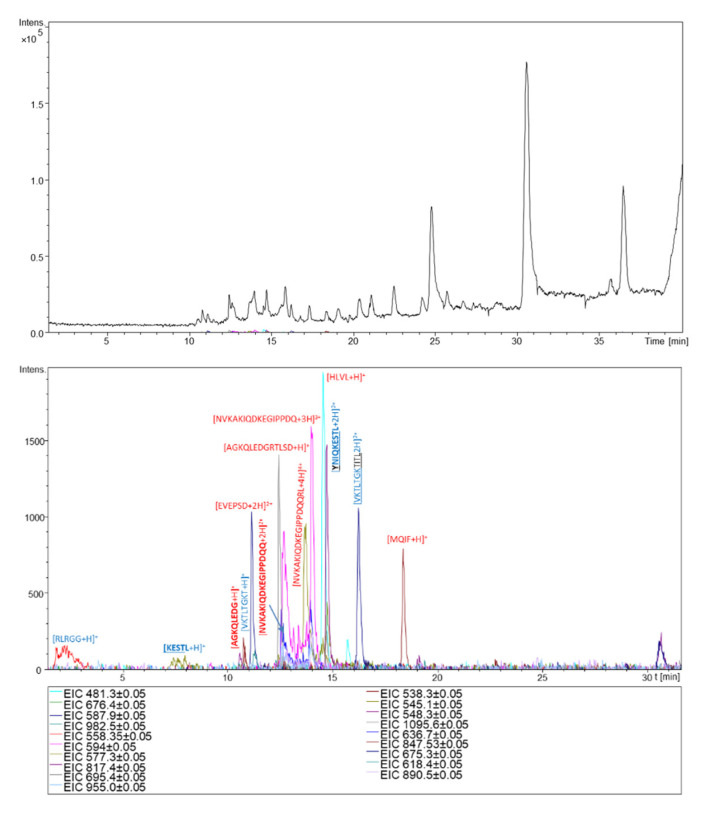
LC-MS analysis of ubiquitin hydrolysate. Total ion current (TIC) (upper panel), zoomed extracted ion chromatograms (EICs) with most abundant signals assigned from 0 to 32 min (middle panel), and found *m*/*z* values (lower panel).

The LC-MS chromatogram of ubiquitin hydrolysate is shown in Figure 13. The black line in the upper panel indicates the total ion chromatogram (TIC). In the performed analysis, most of the peptides were eluted in 10–25 min. The low-intensity peptide signals, indicated with colored lines, are shown in detail within the zoomed part of the chromatogram.

The middle panel in Figure 13 represents the extracted ionchromatograms (EICs) with the most abundant signals identified, while the lower panel shows *m*/*z* values of extracted-ion chromatograms. Sixteen peptides have been identified (other signals correspond to the multiply charged peptides). Most of them elute between 10 and 17 min; however, small hydrophilic peptides such as H-RLRGG-OH or H-KESTL-OH elute earlier, while hydrophobic H-MQIF-OH fragment elutes relatively late (18 min). The most intensive signals within the chromatogram correspond to tetrapeptide H-HLVL-OH and peptides: H-NVKAKIQDKEGIPPDQ-OH, H-YNIQKESTL-OH, and H-AGKQLEDGRTLSD-OH (intensity in the range from 1300 to 2000).

**Figure 14:**
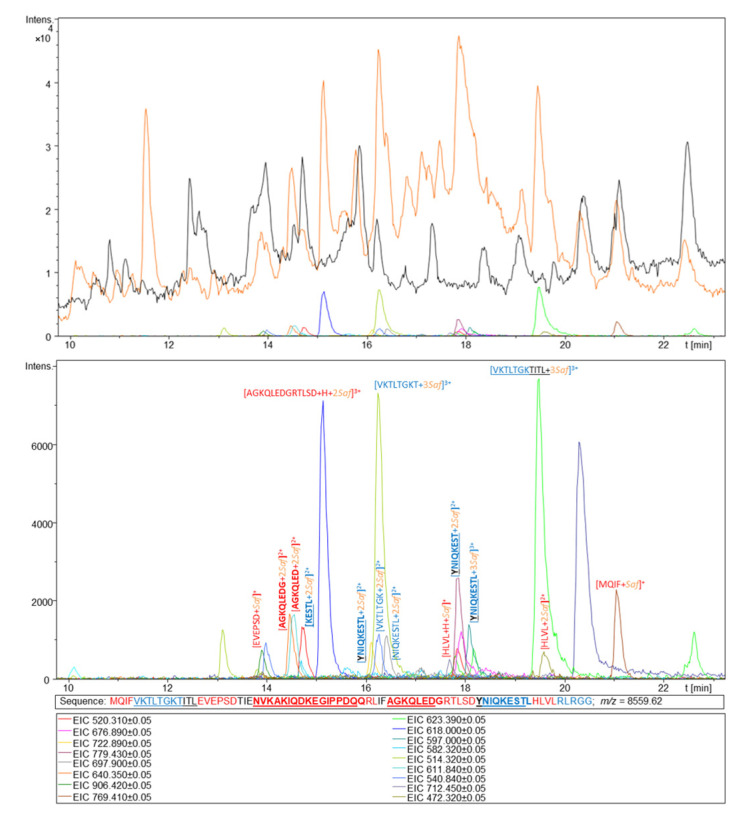
LC-MS analysis of ubiquitin hydrolysate tagged with **5a**: upper panel—TIC, middle panel—zoomed EIC for the most abundant signals assigned from 10 to 24 min; lower panel—ubiquitin sequence with marked hydrolysis products and found *m*/*z*.

Successively, the ubiquitin hydrolysate tagged with *Safirnium P* **5a** has been analyzed. The orange and the colored lines in the LC-MS analysis (Figure 14) indicate total ion current and the extracted ion chromatograms, respectively. The black line indicate TIC of non-tagged ubiquitin hydrolysate. Hence, TIC of tagged peptides as well as the extracted ion chromatograms display significantly higher intensities when compared to the EIC intensities of underivatized ubiquitin hydrolysis products.

The calculated *m*/*z* values for all identified signals of ubiquitin proteolysis products with different charges and different numbers of conjugated **5a** tags are presented in Table 1. Hence, there were 18 charged peptides considered, which have been characterized previously in the LC-MS of ubiquitin hydrolysate with the calculated [M+H]^+^, [M+2H]^2+^, [M+3H]^3+^, or [M+4H]^4+^ *m*/*z* values. The [M+*Saf*]^+^, [M+H+*Saf*]^2+^, [M+2*Saf*]^2+^, [M+2H+*Saf*]^3+^, [M+H+2*Saf*]^3+^, [M+3*Saf*]^3+^, and [M+4Saf]^4+^ values indicate peptide conjugates with diverse charges and the number of conjugated **5a** residues identified in the LC-MS of tagged ubiquitin hydrolysate. All the calculated *m*/*z* values for the ions present in the chromatogram are given in the table. Zoomed extracted ion chromatograms are shown in the middle panel of Figure 14. In general, the majority of peptide conjugates eluted in 14–25 min, which indicates that the *Safirinium P* system slightly increases the hydrophobicity of the analytes. The most abundant signals in the chromatogram were identified. Ester **5a** reacts mainly with peptides that contain lysine residues but also with *N*-terminal free amine groups. The most abundant signals correspond to tagged peptides: H-VKTLTGKTITL-OH and H-VKTLTGKT-OH, which are rich in lysine residues and both conjugated to three *Safirinium P* tags, and H-AGKQLEDGRTLSD-OH, which conjugated to two *Safirinium P* tags. The intensities of these signals are in the range from 7000 to 8000; thus, they are approximately four times higher than the intensities observed for the corresponding non-tagged peptides. In some cases, the observed sensitivity enhancements amount up to eight times based on intensity comparison of EIC signals for free peptides and their tagged analogues.

Finally, the signals of **5a**, its fragmentation products, and hydrolysed ester **3a** were not observed, which indicates that the active ester **5a** reacts efficiently with the ubiquitin hydrolysate.

### 2.4. Fluorescence Microscopy

**Figure 15:**
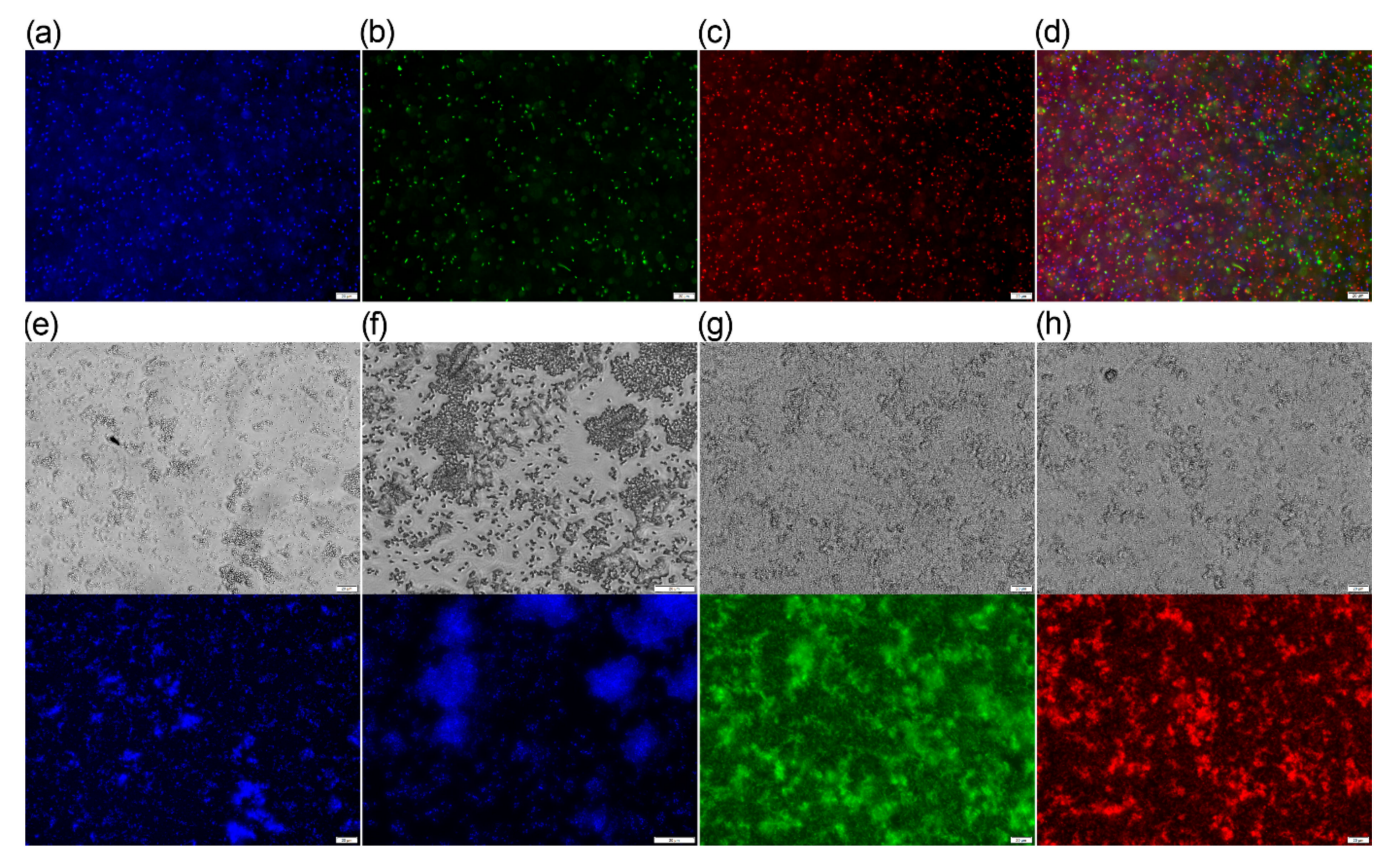
Fluorescence microscopy visualization of free floating bacteria cells in phosphate-buffered saline (PBS) in a mixture of the following strains: (**a**) *E. coli*-pET30 labeled with *Safirinum*, (**b**) *E. coli*-GFP, and (**c**) *E. coli*-DsRed2, (**d**) represents merged pictures. Fluorescence visualization of bacterial biofilm formation by *E. coli* strains producing auto-aggregating protein Ag43 after 6 h of incubation: (**e**,**f**) *E. coli*-pET30 labeled with *Safirinum*, (**g**) *E. coli*-GFP, and (**h**) *E. coli*-DsRed2. In pictures (**e**–**h**), the upper panels represent phase-contrast images of corresponding fluorescence images (lower panels). Scale bars correspond to 20 µm.

Since NHS esters of *Safirinum* form covalent bonds with amine groups, we have tested their usefulness for fast, one-step non-specific labeling of *E. coli* bacteria. After 1 h of incubation of *E. coli*-pET30 bacteria suspended in phosphate-buffered saline (PBS) with *Safirinum* probe **5a** at a concentration of 1 mg/mL the obtained cells pellet indicated fluorescence similar to that observed for strains: *E. coli*-GFP and *E. coli*-DsRed2 labeled with GFP (green fluorescence) and DsRed2 (red fluorescence) proteins, respectively. A lack of fluorescence was evidenced for the control pellet of *E. coli*-pET30 bacteria not incubated with *Safirinum* reagent (Figure S1). In order to perform a better comparison, we have utilized PBS mixture of these three strains to visualize free floating bacteria by means of fluorescence microscopy (Figure 15a–d). Moreover, a long time continuous fluorescence microscopy experiment, i.e., 40 s continuous time lapse video (Video S1) was recorded to estimate the usefulness of *Safirinum* probes for cells labeling. Finally, we have analyzed the biofilm formed by all three bacterial strains after 6 h of incubation at 37 °C by fluorescence microscopy (Figure 15e–h). In all of the above experiments, *Safirinum* derivatization reagent acts as an efficient fluorescence label of bacterial cells with low background emission when analyzed with excitation wavelengths specific for GFP and TRITC (DsRed2) fluorescence (Figure S2). In addition, the bacteria showed the ability to form a biofilm dependent on the presence of the Ag43 surface protein at the comparable level to that of the unstained strain. Hence, this experiment proved that the functionality of Ag43 protein after *Safirinium* staining is not compromised.

## 3. Materials and Methods

### 3.1. General Information

All chemicals were used as received from commercial sources (Acros Organics—Geel, Belgium, Alfa-Aesar—Ward Hill, MA, USA, or Sigma-Aldrich—Darmstadt, Germany) and used without further purification. All nuclear magnetic resonance (NMR) spectra were recorded on a Varian Mercury-VX 300 MHz spectrometer at 25 °C. Chemical shifts (δ) are given in parts per million (ppm) and internally referenced to solvent signals. Coupling constants (*J*) are reported in Hertz (Hz). Splitting patterns are designated as s (singlet), bs (broad singlet), d (doublet), t (triplet), or m (multiplet). The IR (KBr pellets) spectra were recorded on a Thermo Scientific Nicolet 380 FT-IR spectrometer. Melting points were determined on an X-4 melting point apparatus with a microscope and were uncorrected. Analytical reverse-phase high-performance liquid chromatography with mass spectrometry detection (RP-HPLC-MS) experiments were performed on Agilent 1200 chromatograph, equipped with the UV detector—210 and 280 nm and coupled with micrOTOF-Q mass spectrometer in positive electrospray ionization (ESI) mode (described above) and SHIMADZU (Kyoto, Japan) LCMS-2020 EV mass spectrometer. The separation was carried on a reversed phase column: Aeris Peptide, Phenomenex XB-C18 (50 × 2.1 mm, 100 Å, 3.6 μm), flow—0.2 mL/min, injection volume—5 μL, eluents: A = H2O + 0.1% HCOOH, B = 80% MeCN in H2O + 0.1% HCOOH. Analytical HPLC of derivatized peptide **8** was carried on Thermo Separation system with UV detection (210 nm) and a YMC-Pack RP C18 column (4.6 × 250 mm, 5 μm), flow—1 mL/min, injection volume—100 μL, eluents: A = H_2_O + 0.1% TFA; B = 80% MeCN in H_2_O + 0.1% TFA. The MS and MS/MS experiments were performed on Fourier Transform Ion Cyclotron Resonance (FT-ICR) Apex-Qe Ultra 7T instrument (Bruker Daltonics, Brema, Germany) or micrOTOF-Q instrument (Bruker Daltonics, Brema, Germany) with ESI ionization in positive ion mode, *m*/*z* range: 100–2200, drying gas—N_2_ with the flow 4 L/min, internal capillary temperature—200 °C, voltage—4500 V, eluents: MeCN:H_2_O:HCOOH (50:50:0,1; *v/v/v*), with the flow—3 μL/min. The specified and set resolution for microTOF-Q was 15,000 FWHM. For FT-ICR, the specified resolution was 1,000,000 FWHM, although the set one was 100,000 FWHM. For MS/MS, the parent ions were fragmented by collision-induced dissociation (CID) with argon as the collision gas. The collision energy was optimized for the best fragmentation efficiency. The mass spectra analyses were performed using Compass DataAnalysis 4.0 (Bruker, Bremen, Germany) software. Samples for lyophilization were frozen in liquid nitrogen and lyophilized on a Speedvac or Freeze-dryer from Labconto (Kansas City, MO, USA). The absorption and emission spectra of *Safirinium* dyes and their derivatives have been presented previously [42,43,48].

### 3.2. Chemical Synthesis

Isoxazolones **1** and **2**, 2,2-dialkyl-[1,2,4]triazolo[4,3-*a*]pyridin-2-ium-8-carboxylates **3a,c** and 2,2-dialkyl-[1,2,4]triazolo[4,3-*a*]quinolin-2-ium-4-carboxylates **4a-c**, as well as NHS esters **5a** and **6a** were synthesized according to our previously reported procedures [42,43,48,55]. Synthesis and characterization of conjugate **7a** was also presented earlier in the literature [42].

#### 3.2.1. Synthesis of 8-Carboxy-5,7-Dimethyl-2,2-Dioctyl-2,3-Dihydro-[1,2,4]Triazolo[4,3-a]Pyridin-2-ium Chloride (3b)

Isoxazolone (0.354 g, 1.9 mmol) was dissolved in 10 mL of methanol; then, dioctylamine (0.574 mL, 1.9 mmol) and 35% water solution of formaldehyde (0.598 mL, 7.6 mmol) were added. The reaction mixture was stirred for 16 h at room temperature (progress of the reaction was monitored by LC-MS) and subseqently evaporated under reduced pressure. The resulted solid was washed with acetone (2 × 3 mL) and quantitatively converted into hydrochloride form with use of methanolic HCl solution. Yield: 43% (0.371 g); m.p. 130–131 °C; ^1^H NMR (300 MHz, CDCl_3_) (Figure S3): δ = 0.83 (t, *J* = 6.8 Hz, 6H, CH_3_), 1.21–1.26 (m, 20H, CH_2_), 1.70–1.86 (m, 4H, CH_2_), 2.22 (s, 3H, CH_3_), 2.34 (s, 3H, CH_3_), 3.51–3.57 (m, 2H, CH_2_), 3.76–3.83 (m, 2H, CH_2_), 4.12 (bs, 1H, OH), 5.75 (s, 1H, CH), 6.21 (s, 2H, CH_2_); ^13^C NMR (75 MHz, CDCl_3_) (Figure S4): δ = 14.0, 18.9, 20.2, 22.5, 22.9, 26.3, 29.2 (2 signals), 31.6, 66.6, 73.6, 110.9, 117.7, 138.9, 147.5, 156.2, 168.4; IR (KBr): 3394, 3003, 2953, 2926, 2855, 1665, 1638, 1589, 1562, 1532, 1468, 1376, 1355, 1185, 803, 606 cm^-1^; MS (ESI) *m*/*z*: 418 [M+1]^+^.

#### 3.2.2. Synthesis of Reactive Safirinium P (5b,c) and Q (6b,c) Probes

Compounds **5b,c** and **6b,c** were synthesized similarly to our previously reported procedures with only minor modifications [49]. The corresponding *Safirinium* dye **3a-c** or **4a-c** (0.350 mmol) was dissolved in 7 mL of anhydrous dimethylformamide (DMF), then *N*,*N*-diisopropylcarbodiimide (DIC) (0.082 mL, 0.525 mmol), and NHS (0.060 g, 0.525 mmol) were added. The reaction mixture was sonicated for 15 min and stirred for 24 h at room temperature. The progress of the reaction was monitored with LC-MS. Subsequently to complete conversion of the starting material, the reaction mixture was evaporated under reduced pressure. Compounds **5b** and **6b** were purified by crystallization from methanol/acetone mixture (1:20, *v*/*v*). The resulted solid of crude compound **5c** was dissolved in water (3 mL), whereupon the solution was filtrated and evaporated to dryness. The solid residue was washed with acetone (3 × 2 mL). In the case of compound **6c**, which is susceptible to hydrolysis, the resulted crude solid was washed with acetone (3 × 2 mL) and crystalized from methanol/acetone mixture (1:20, *v*/*v*) prior to characterization.

8-(((2,5-Dioxopyrrolidin-1-yl)oxy)carbonyl)-5,7-dimethyl-2,2-dioctyl-2,3-dihydro-[1,2,4]triazolo[4,3-*a*]pyridin-2-ium chloride (**5b**)

Obtained from 0.159 g of **3b**. Yield: 94% (0.181 g); m.p. 88–89 °C; ^1^H NMR (300 MHz, CDCl_3_) (Figure S5): δ = 0.81 (t, *J* = 6.8 Hz, 6H, CH_3_), 1,20–1,27 (m, 20H, CH_2_), 1.76–1.81 (m, 4H, CH_2_), 2.47 (s, 3H, CH_3_), 2.83 (s, 3H, CH_3_), 2.84 (s, 4H, CH_2_), 3.50–3.58 (m, 2H, CH_2_), 3.86–3.96 (m, 2H, CH_2_), 5.93 (s, 1H, CH), 6.45 (s, 2H, CH_2_); ^13^C NMR (75 MHz, CDCl_3_) (Figure S6): δ = 14.0, 20.3, 22.5, 22.6, 22.8, 25.7, 26.1, 29.1 (2 signals), 31.6, 67.5, 73.0, 101.5, 111.7, 148.2, 155.4, 159.2, 160.2, 168.8; IR (KBr): 3422, 2955, 2927, 2856, 1769, 1742, 1625, 1559, 1362, 1200, 1142, 1116, 1062, 994, 645 cm^−1^; MS (ESI) *m*/*z*: 515 [M]^+^.

8-(((2,5-Dioxopyrrolidin-1-yl)oxy)carbonyl)-5,7-dimethyl-3*H*-spiro[[1,2,4]triazolo[4,3-*a*]pyridine-2,1′-pyrrolidin]-1′-ium chloride (**5c**)

Obtained from 0.100 g of **3c**. Yield: 85% (0.114 g); m.p. 72–75 °C; ^1^H NMR (300 MHz, CD_3_OD) (Figure S7): δ = 2.26–2.40 (m, 4H, CH_2_), 2.43 (s, 3H, CH_3_), 2.51 (s, 3H, CH_3_), 2.89 (s, 4H, CH_2_), 3.91 (t, *J* = 5.3 Hz, 4H, CH_2_), 5.96 (s, 2H, CH_2_), 6.26 (s, 1H, CH); ^13^C NMR (75 MHz, CD_3_OD) (Figure S8): δ = 17.9, 21.3, 24.9, 25.2, 68.7, 74.3, 101.5, 111.4, 148.3, 155.4, 160.1, 170.2, 173.5; IR (KBr): 3423, 2968, 2938, 2818, 1736, 1713, 1624, 1562, 1209, 1066, 995, 650 cm^−1^; MS (ESI) *m*/*z*: 345 [M]^+^.

4-(((2,5-Dioxopyrrolidin-1-yl)oxy)carbonyl)-2,2-dioctyl-1,2-dihydro-[1,2,4]triazolo[4,3-*a*]quinolin-2-ium chloride (**6b**)

Obtained from 0.166 g of **4b**. Yield: 94% (0.190 g); m.p. 115–118 °C; ^1^H NMR (300 MHz, CDCl_3_): δ = 0.78 (t, 6H, CH_3_), 1.11–1.23 (m, 20H, CH_2_), 1.76–1.79 (m, 4H, CH_2_), 2.57 (s, 2H, CH_2_), 2.91 (s, 2H, CH_2_), 3.63–3.84 (m, 4H, CH_2_), 6.17 (s, 2H, CH_2_), 7.23 (t, *J =* 8.1 Hz, 1H, CH), 7.44 (d, *J =* 8.1 Hz, 1H, CH), 7.56 (d, *J =* 8,1 Hz, 1H, CH), 7.61 (t, *J =* 8.1 Hz, 1H, CH), 8.33 (s, 1H, CH); ^13^C NMR (75 MHz, CDCl_3_): δ = 14.0, 22.5, 23.4, 26.1, 29.1, 29.2, 31.6, 42.1, 67.3, 71.9, 114.54, 117.3, 120.3, 124.0, 130.4, 134.2, 134.8, 154.4, 157.9, 164.9, 172.9; IR (KBr): 3341, 2958, 2925, 2855, 1741, 1711, 1619, 1572, 1549, 1459, 1213, 1169, 760, 650 cm^−1^; MS (ESI) *m*/*z*: 537 [M]^+^.

4-(((2,5-Dioxopyrrolidin-1-yl)oxy)carbonyl)-1*H*-spiro[[1,2,4]triazolo[4,3-*a*]quinoline-2,1′-pyrrolidin]-1′-ium chloride (**6c**)

Obtained from 0.107 g of **4c**. Yield: 78% (0.110 g); m.p. 184–189 °C; IR (KBr): 3341, 3112, 2968, 2942, 2877, 1767, 1708, 1621, 1571, 1554, 1459, 1210, 1196, 1164, 1133, 1088, 1071, 1057, 997, 775, 761, 648 cm^-1^; MS (ESI) *m*/*z*: 367 [M]^+^.

#### 3.2.3. Synthesis of Peptide Conjugates 7a-c

Reactive ester (**5a-c**) (0.1 mmol) and lysine-containing peptide Ac-AKF-NH_2_ (0.040 g, 0.1 mmol) were dissolved in 3 mL of DMF. The reaction mixture was stirred for 16 h at room temperature. The progress of the reaction was monitored by LC-MS. When the reaction was finished, the mixture was evaporated and washed with acetone (3 × 1 mL). Compound **7a** has been reported previously [42].

8-(((*S*)-5-((*S*)-2-acetamidopropanamido)-6-(((*S*)-1-amino-1-oxo-3-phenylpropan-2-yl)amino)-6-oxohexyl)carbamoyl)-5,7-dimethyl-3*H*-spiro[[1,2,4]triazolo[4,3-*a*]pyridine-2,1′-pyrrolidin]-1′-ium chloride (**7b**): HRMS (ESI) *m*/*z*: calc. 805.5698, found 805.5525 [M]^+^.

8-(((*S*)-5-((*S*)-2-acetamidopropanamido)-6-(((*S*)-1-amino-1-oxo-3-phenylpropan-2-yl)amino)-6-oxohexyl)carbamoyl)-5,7-dimethyl-2,2-dioctyl-2,3-dihydro-[1,2,4]triazolo[4,3-*a*]pyridin-2-ium chloride (**7c**): HRMS (ESI) *m*/*z*: calc.635.3664, found 635.3663 [M]^+^.

The peptide conjugate **7a** was subjected to hydrogen–deuterium exchange. To the 10 μL of 2 mM solution of **7a** in water 10 μL of triethylamine (TEA) in 980 μL of D_2_O was added and subsequently incubated for 24 h at room temperature. Next, upon lyophilization, the sample was re-dissolved in water and incubated for up to 5 days for back-exchange. All the samples of both non-deuterated and deuterated analogues were analyzed by ESI-FT-ICR-MS and MS/MS.

#### 3.2.4. Synthesis of the Derivatized Peptide 8

A model Ac-AAK(Mtt) sequence for on-resin tagging was synthetized manually using standard Fmoc-solid-phase-peptide-synthesis (Fmoc-SPPS) procedure on Fmoc-Lys(Mtt)-Wang resin (loading 0.58 mmol/g). Orthogonal protection of the lysine side chain by the methyltrityl (Mtt) group was applied, and it was deprotected by 1% trifuoroacetic acid (TFA)/dichloromethane (DCM). Ac-AAAK-Wang was treated on-resin with **5a**. For this purpose, 500 mg of the peptydylresin was added to a polypropylene syringe with a sinter (Intavis) and swelled for 30 min in DMF. Then, 198 mg of **5a** (0.58 mol—2 eq.) dissolved in 4 mL of DMF was added, and the mixture was stirred for 24 h at the rotator. When the reaction was complete, as confirmed by negative Kaiser test, the resin was washed by the following mixtures: 3×DMF, DMF/dichloromethane (DCM, 50/50), 3×DCM, DCM/MeOH (50/50), 3×MeOH and dried in the dessicator. The tagged peptide **8** was released from the resin using the standard procedure with 2 mL of TFA/H_2_O/triisopropylsilane (TIS, 95:2.5:2.5 *v*/*v*/*v*) and stirred for 2 h at the rotator. The mixture was evaporated, and the residue was dissolved in water and lyophilised. The obtained crude product **8** (71.1 mg) was dissolved in 1.5 mL of water and purified by preparative RP-HPLC on the Varian ProStar instrument (Palo Alto, CA, USA) equipped with the UV detector—210 and 280 nm, column TSKgel ODS-120T (215 × 30 mm, 150 Å, 10 μm), flow—7 mL/min, eluents: A = H_2_O + 0.1% trifuoroacetic acid (TFA), B = 80% MeCN / H_2_O + 0.1% TFA, gradient 15–30% B in 40 min. Purified tagged peptide was subjected to further analytical HPLC (Thermo, gradient 0–80% B in 40 min) (Figure S9), MS and MS/MS analysis. Rt = 14.51 min (HRMS (ESI) *m*/*z*: calc. 633.3719, found 633.3674 [M]^+^.

### 3.3. Tagging the Ubiquitin Hydrolysate with 5a

Ubiquitin pepsine hydrolysate (1 mg, 5.78 × 10^−7^ mol) was dissolved in 1 mL of triethylammonium bicarbonate (TEAB) buffer (pH 8.4–8.6). The solution was divided into two portions: 0.5 mL was retained as a control sample and the second 0.5 mL was tagged with 1.13 mg (57 eq.) of **5a** dissolved in 113 μL of TEAB and incubated for 1 h. Water (0.7 mL) was added to hydrolyze the unreacted active ester. Both samples of protein hydrolysates, derivatized and non-derivatized, were evaporated on a speedvac. Subsequently, they were diluted 50 times with water and analyzed by LC-MS. The LC-MS analysis was performed on micrOTOF-Q coupled with the analytic HPLC Agilent 1200, equipped with the UV detector—210 and 280 nm, RP column: Aeris Peptide, Phenomenex XB-C18 (50 × 2.1 mm, 100 Å, 3.6 μm), flow—0.2 mL/min, injection volume—5 μL, eluents: A = H_2_O + 0.1% HCOOH, B = 80% MeCN in H_2_O + 0.1% HCOOH with the gradient: 0–5% B in 3 min, 5–60% B in 55 min., 60–100% B in 40 min. The separation was performed at room temperature (22 °C).

### 3.4. Fluorescence Microscopy of Bacteria

The *E. coli* Top10 bacteria (Invitrogen, Carlsbad, CA, USA) were transformed with the following plasmids: *pSFOXB20-daGFP*, pDsRed2 and pET30, giving strains: *E. coli*-GFP, *E. coli*-DsRed2, and *E. coli*-pET30. pSFOXB20-daGFP (Oxford Genetics, Oxford, UK) is a vector encoding GFP (Green Fluorescent Protein) controlled by the OXB20 constitutive promoter. The plasmid possesses an origin of replication derived from pBR322 and a kanamycin resistance gene. It was used to visualize the bacterial cells as green fluorescent using fluorescence microscopy. pDsRed2 (Takara, Saint-Germain-en-Laye, France) is a plasmid encoding DsRed2 (red fluorescent protein) controlled by the *lac* promoter. The plasmid is the derivative of pUC19 and encodes an ampicillin resistance gene. It was used to visualize the bacterial cells as red fluorescent. pET30 (Novagen-Merck, Darmstadt, Germany) plasmid possesses an origin of replication derived from pBR322 and a kanamycin resistance gene. This strain was labeled with *Safirinum* dye **5a** and visualized by means of blue fluorescent staining. All used bacterial strains were cultivated overnight with agitation of 160 rpm at 37 °C in 50 mL of Luria broth (LB) supplemented with the appropriate antibiotics (100 μg ampicillin mL^−1^ and 20 μg kanamycin mL^−1^; Sigma-Aldrich). Overnight cultures were centrifuged and suspended in phosphate-buffered saline (PBS) to an optical density of OD_600_ = 0.5. For fluorescence microscopy, the *E. coli*-pET30 strain suspended in PBS was incubated with dye **5a** at the final concentration of 1 mg/mL for 60 min at room temperature. Then, the non-bound dye was removed by a five-fold exchange of PBS buffer by consecutive centrifugation and suspension steps. Finally, the *Safirinum* labeled *E. coli*-pET30 bacteria were adjusted with PBS to OD_600_ = 0.5.

In the first type of experiments, equal volumes of all three bacterial strains were mixed. Then, 3 mL of the suspension was placed in 35 mm polystyrene dishes (Corning, New York, NY, USA), and free floating bacteria were visualized by fluorescence microscopy. In the second type of experiments, 3 mL of each bacterial strain was inoculated in a separate dish and incubated at 37 °C for 6 h. After this time, the formation of bacterial biofilm at the bottom of the dish was visualized using fluorescence microscopy. Each type of experiment was repeated three times.

The free floating or biofilm-forming bacteria emitting appropriate fluorescence were recorded with the Olympus IX73 inverted fluorescence microscope equipped with the UCPlanFL N 20×/0.70 or LUCPlanFL N 40×/0.60 objectives, a Olympus U-HGLGPS fluorescence light source, and a Hamamatsu Orcaflesh 2.8 CMOS digital camera. For blue, green, and red fluorescence, the following filter sets were used, respectively: DAPI HC BP, GFP-1828A, and TRITC-B-000 (all from Semrock, IDEX Corporation (Lake Forest, IL, USA). Single frame images and time-lapse digital videos were recorded using the Olympus cellSens Dimension 1.18 software. The time-lapse videos were converted to the mp4 format using Kdenlive.

## 4. Conclusions

*Safirinium* NHS active esters have been found useful in bacteria bioimaging applications and enhancement of peptide signals in ESI-MS analyses. The preparation of the enhancers is inexpensive and simple. The developed *Safirinium P* tags can be applied in solid phase peptide synthesis, which is now the most common and efficient way for the preparation of peptides, and the resulting labeled product is stable in the course of peptide cleavage using 95% TFA. The *Safirinium* labels undergo transformations during CID experiments, including Hofmann elimination, homolytic cleavage, and charge remote fragmentation, which limits their application and results in a characteristic peak pattern. Although the described compounds do not allow for neat peptide sequencing, the presented data proved that they react efficiently with both ε-amine groups of lysine and *N*-terminal amine groups of peptides. Thus, the introduction of these fluorescent tags [42], that bear quaternary ammonium salt (QAS) moiety, enables the effective detection of such labeled peptides by means of both fluorescence and mass spectrometry. When compared to the analysis of ubiquitin hydrolysate performed by Jaremko et al. [54], the protein sequence coverage is very good and reaches 80%. The *Safirinium* system enhances the intensity of MS signals up to eight times, which confirms its potential applications in ionization tagging and sensitive detection of analyte signals, especially of lysine-rich peptides. This may lead to the discovery of new biomarkers based on proteins of low abundance; therefore, the examined systems could find application as ionization enhancers. Furthermore, cell labeling experiments on *E. coli* Top10 bacteria revealed that the studied probes can stand the challenging environment of biological systems such as living cell cultures, and the fluorescent visualization of free-floating bacteria cells with *Safirinium P* dyes gives comparable results to those obtained for bacteria labeled with GFP (green fluorescence) and DsRed2 (red fluorescence) proteins.

## Figures and Tables

**Table 1:** The calculated *m*/*z* for identified ubiquitin fragments and their conjugates with **5a** ionization tag (*Saf*).

No	Peptide Sequence	[M+H]^+^	[M+*Saf*]^1+^	[M+2H]^2+^	[M+H+*Saf*]^2+^	[M+2*Saf*]^2+^	[M+3H]^3+^	[M+2H+*Saf*]^3+^	[M+H+2*Saf*]^3+^	[M+3*Saf*]^3+^	[M+4H]^4+^	[M+4*Saf*]^4+^
1	NVKAKIQDKEGIPPDQQRL		-	-	-	-	726.4061	-	-	-	545.0564	-
2	NVKAKIQDKEGIPPDQQ		-	954.5129	-	-	-	-	-	-	-	-
3	NVKAKIQDKEGIPPDQ		-	890.4837	-	-	-	-	-	-	-	676.8827
4	AGKQLEDGRTLSD		-	695.3521	-	926.4893	-	540.9496	617.9953	-	-	
5	VKTLTGKTITL		-	587.8740	-	-	-	-	-	623.3889	-	-
6	YNIQKESTL	1095.5681	-	548.2877	-	779.4249	-	-	-	596.9981	-	-
7	YNIQKEST	982.4840	-	-	-	722.8829	-	-	-	-	-	-
8	NIQKESTL	-	-	-	582.3246	697.8932	-	-	-	-	-	-
9	VKTLTGKT	847.5248	-	424.2661	-	-	-	-	-	514.3170	-	-
10	AGKQLEDG	817.4051	-	-	-	640.3434	-	-	-	-	-	-
11	AGKQLED	-	-	-	-	611.8326	-	-	-	-	-	-
12	QRLIF	676.4141	-	-	-	-	-	-	-	-	-	-
13	EVEPSD	675.2832	906.4204	-	-	-	-	-	-	-	-	-
14	VKTLTG	618.3821	-	-	-	540.8319	-	-	-	-	-	-
15	KESTL	577.3192	-	-	-	520.3004	-	-	-	-	-	-
16	RLRGG	558.3471	-	-	-	-	-	-	-	-	-	-
17	MQIF	538.2694	769.4066	-	-	-	-	-	-	-	-	-
18	HLVL	481.3133	712.4505	-	-	472.2975	-	-	-	-	-	-

## Supplementary Materials

Supplementary materials can be found at https://www.mdpi.com/1422-0067/21/24/9643/s1.

## Abbreviations

CID	collision-induced dissociation
DCM	dichloromethane
EIC	extracted ion chromatogram
ESI	electrospray ionization
FT-ICR	Fourier-Transform Ion-Cyclotrone-Resonance
DIC	*N*,*N*-diisopropylcarbodiimide
DMF	dimethylformamide
DHX	deuterium-hydrogen-exchange
GFP	green fluorescence protein
HDX	hydrogen-deuterium exchange
HPLC	high performance liquid chromatography
ITs	ionization tags
LB	Luria broth
LC	liquid chromatography
MRM	multiple reaction monitoring
MS	mass spectrometry
MS/MS	tandem mass spectrometry
NHS	*N*-hydroxysuccinimide
NMR	nuclear magnetic resonance
OD	optical density
Oct	octyl
PAGE	polyacrylamide gel electrophoresis
PBS	phosphate-buffered saline
QAS	quaternary ammonium salts
RP	reverse-phase
TEA	triethylamine
TEAB	triethylammonium bicarbonate
TFA	trifuoroacetic acid
TIC	total ion chromatogram
TIS	triisopropylsilane
TRITC	tetramethylrhodamine-isothiocyanate
