# Supplementary material for: Application of Safirinium N-Hydroxysuccinimide Esters to Derivatization of Peptides for High-Resolution Mass Spectrometry, Tandem Mass Spectrometry, and Fluorescent Labeling of Bacterial Cells
Source: Int J Mol Sci. 2020 Dec 17;21(24):9643. doi: 10.3390/ijms21249643 (PMC7767236; doi:10.3390/ijms21249643)
Supplement: Supplementary file 1 [file ijms-21-09643-s001.zip › ijms-1028791 SI done/ijms-1028791 SI.pdf]

Supplementary materials

Application of *Safirinium N*-Hydroxysuccinimide Esters to Derivatization of Peptides for High-Resolution Mass Spectrometry, Tandem Mass Spectrometry, and Fluorescent Labeling of Bacterial Cells

Joanna Fedorowicz [1,*,†], Magdalena Wierzbicka [2,†], Marek Cebrat [2], Paulina Wiśniewska [3], Rafał Piątek [4], Beata Zalewska-Piątek [4], Zbigniew Szewczuk [2] and Jarosław Sączewski [3]

1 Department of Chemical Technology of Drugs, Faculty of Pharmacy, Medical University of Gdańsk, Al. Gen. J. Hallera 107, 80-416 Gdańsk, Poland
2 Faculty of Chemistry, University of Wrocław, ul. F. Joliot-Curie 14, 50-383 Wrocław, Poland; magdalena.wierzbicka@chem.uni.wroc.pl (M.W.); marek.cebrat@chem.uni.wroc.pl (M.C.); zbigniew.szewczuk@chem.uni.wroc.pl (Z.S.)
3 Department of Organic Chemistry, Faculty of Pharmacy, Medical University of Gdańsk, Al. Gen. J. Hallera 107, 80-416 Gdańsk, Poland; pauwis93@gumed.edu.pl (P.W.); js@gumed.edu.pl (J.S.)
4 Department of Molecular Biotechnology and Microbiology, Chemical Faculty, Gdańsk University of Technology, Narutowicza 11/12, 80-233 Gdańsk, Poland; rafpiate@pg.edu.pl (R.P.); beazalew@pg.edu.pl (B.Z.-P.)
* Correspondence: jfedorowicz@gumed.edu.pl; Tel.: +48-58-349-1957
† These authors contributed equally to this work.

Received: 21 November 2020; Accepted: 15 December 2020; Published: date

Table of Contents:

Figure S1. UV-light induced fluorescence of bacterial pellets of following strains: (a) *E. coli*-pET30 labeled with *Safirinum*, (b) *E. coli*-GFP, (c) *E. coli*-DsRed2 and (d) non-labeled *E. coli*-pET30.

Figure S2. Visualization of floating *E. coli*-pET30 bacteria labeled with *Safirinum* **5a** using (a) phase-contrast microscopy and fluorescence microscopy with filter sets dedicated to: (b) DAPI (blue fluorescence), (c) GFP (green fluorescence) and (d) TRITC (red fluorescence). Scale bars correspond to 20 μm.

Figure S3. ^{1}H-NMR spectrum of **3b**

Figure S4. ^{13}C-NMR spectrum of **3b**

Figure S5. ^{1}H-NMR spectrum of **5b**

Figure S6. ^{13}C-NMR spectrum of **5b**

Figure S7. ^{1}H-NMR spectrum of **5c**

Figure S8. ^{13}C-NMR spectrum of **5c**

Figure S9. HPLC chromatogram of **8**

Video S1. Representative time lapse videos of PBS suspension of E. coli-pET30 bacteria labelled with *Safirinum* **5a** visualized by phase-contrast and fluorescence microscopy. The accelerated video corresponds to 2.5 sec. of phase contrast and 40 sec. of fluorescence original records. The focus was set to the dish bottom.

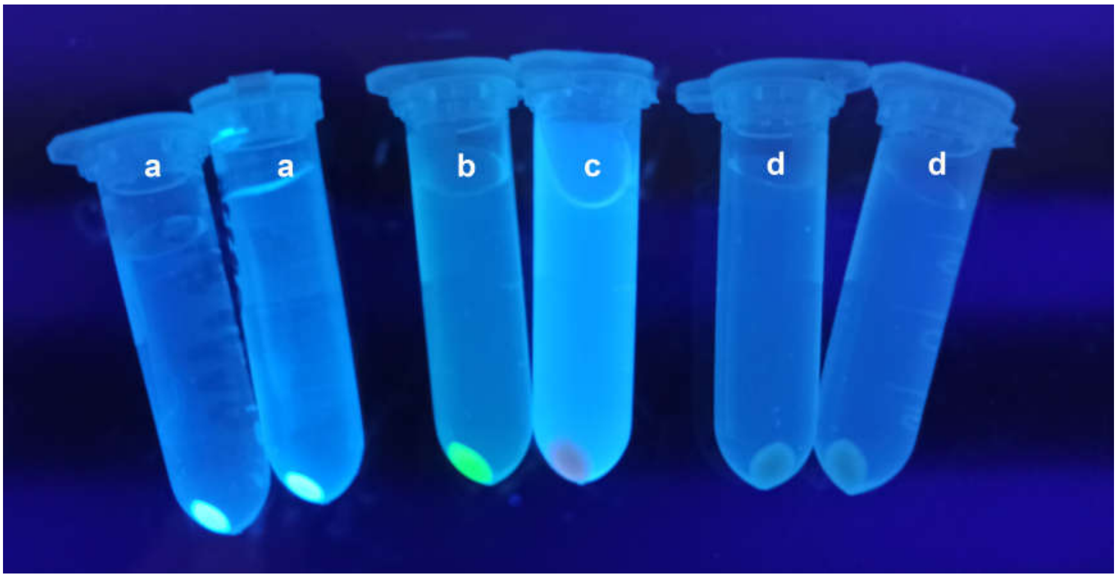

Figure S1. UV-light induced fluorescence of bacterial pellets of following strains: (a) *E. coli*-pET30 labeled with *Safirinum* **5a**, (b) *E. coli*-GFP, (c) *E. coli*-DsRed2 and (d) non-labeled *E. coli*-pET30.

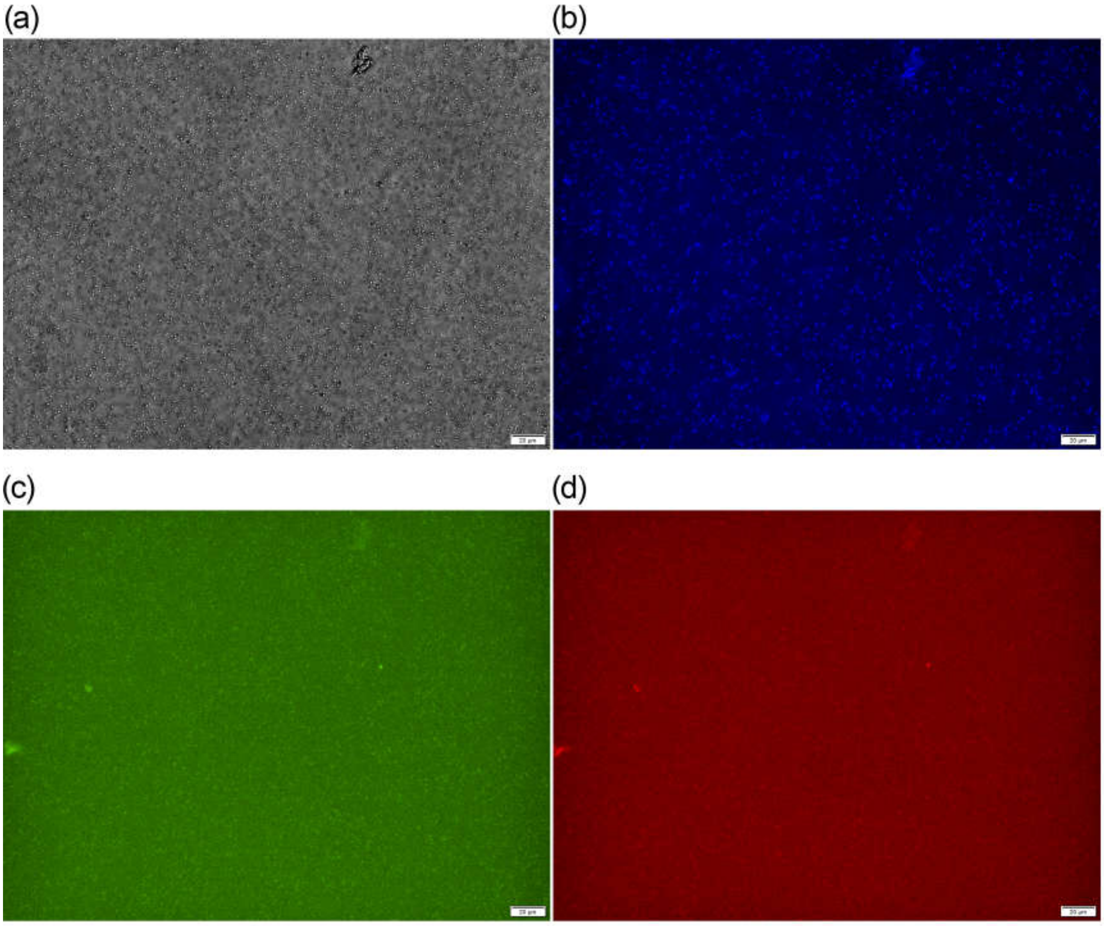

Figure S2. Visualization of floating *E. coli*-pET30 bacteria labeled with *Safirinum* **5a** using (a) phase-contrast microscopy and fluorescence microscopy with filter sets dedicated to: (b) DAPI (blue fluorescence), (c) GFP (green fluorescence) and (d) TRITC (red fluorescence). Scale bars correspond to 20 μm.

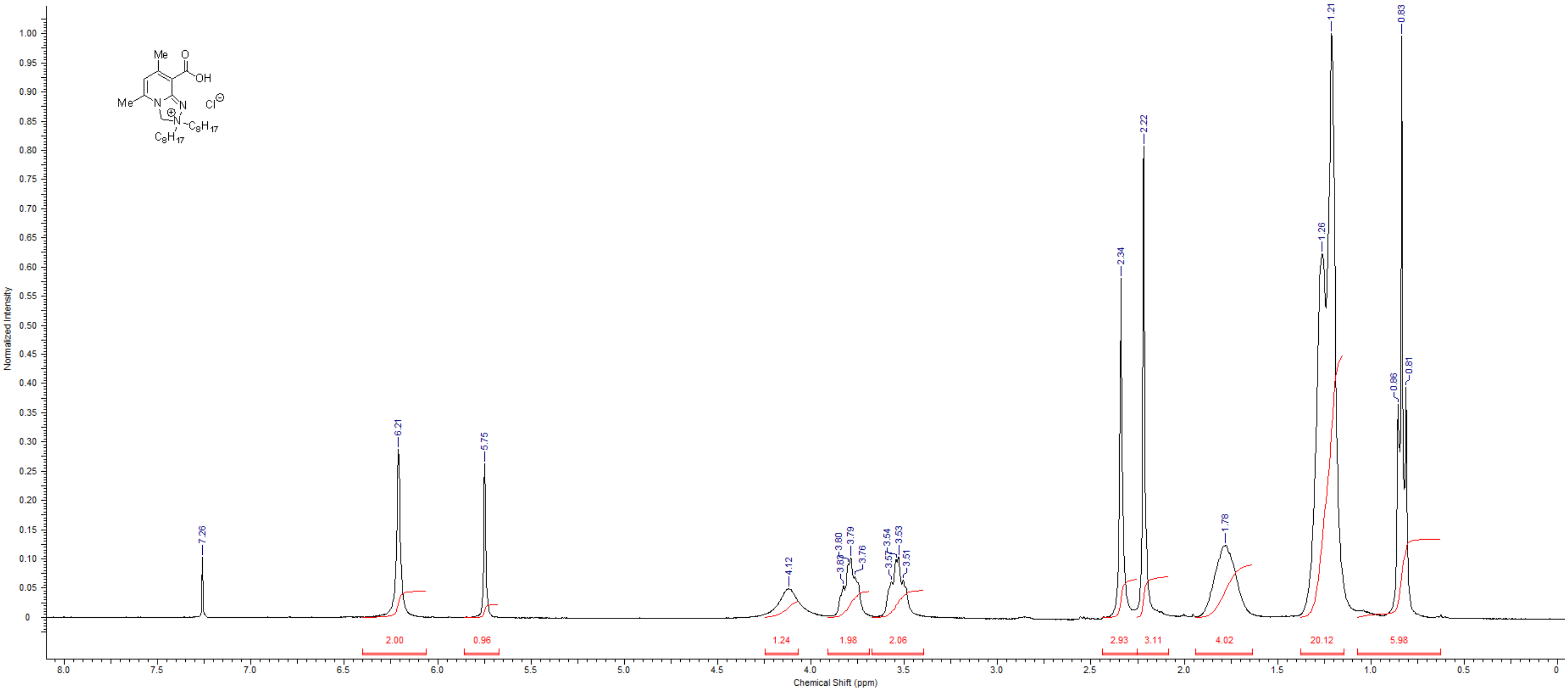

Figure S3. ^{1}H-NMR spectrum of **3b**

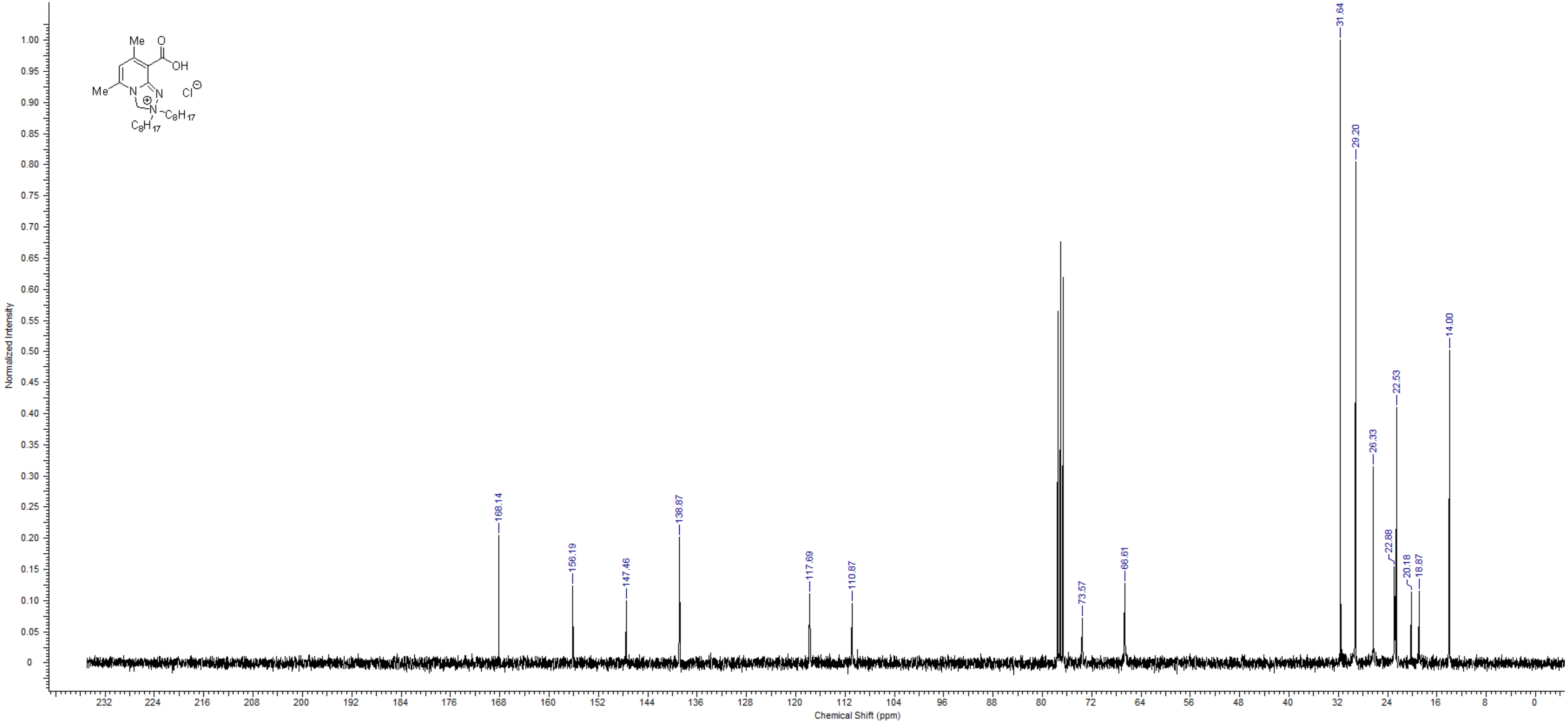

Figure S4. ^{13}C-NMR spectrum of **3b**

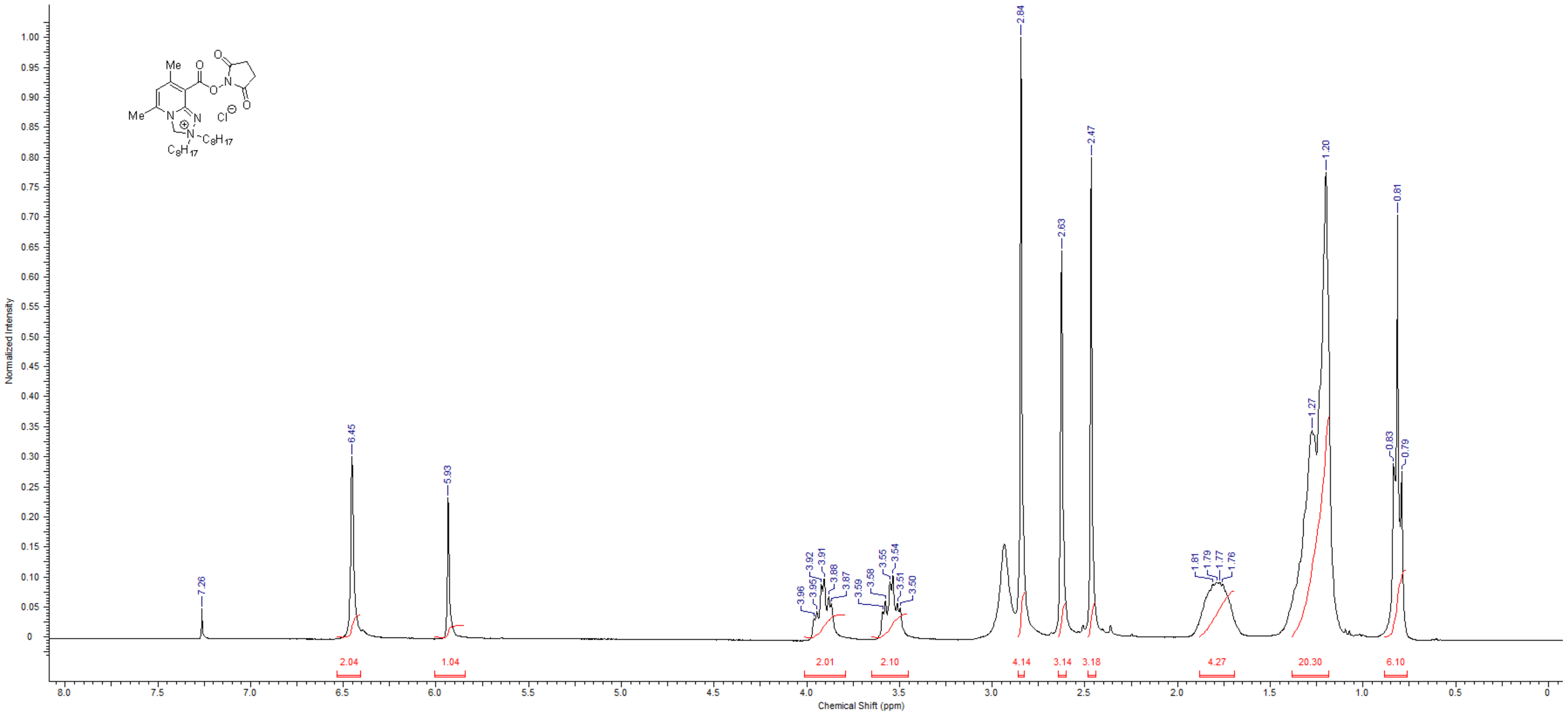

Figure S5. ^{1}H-NMR spectrum of **5b**

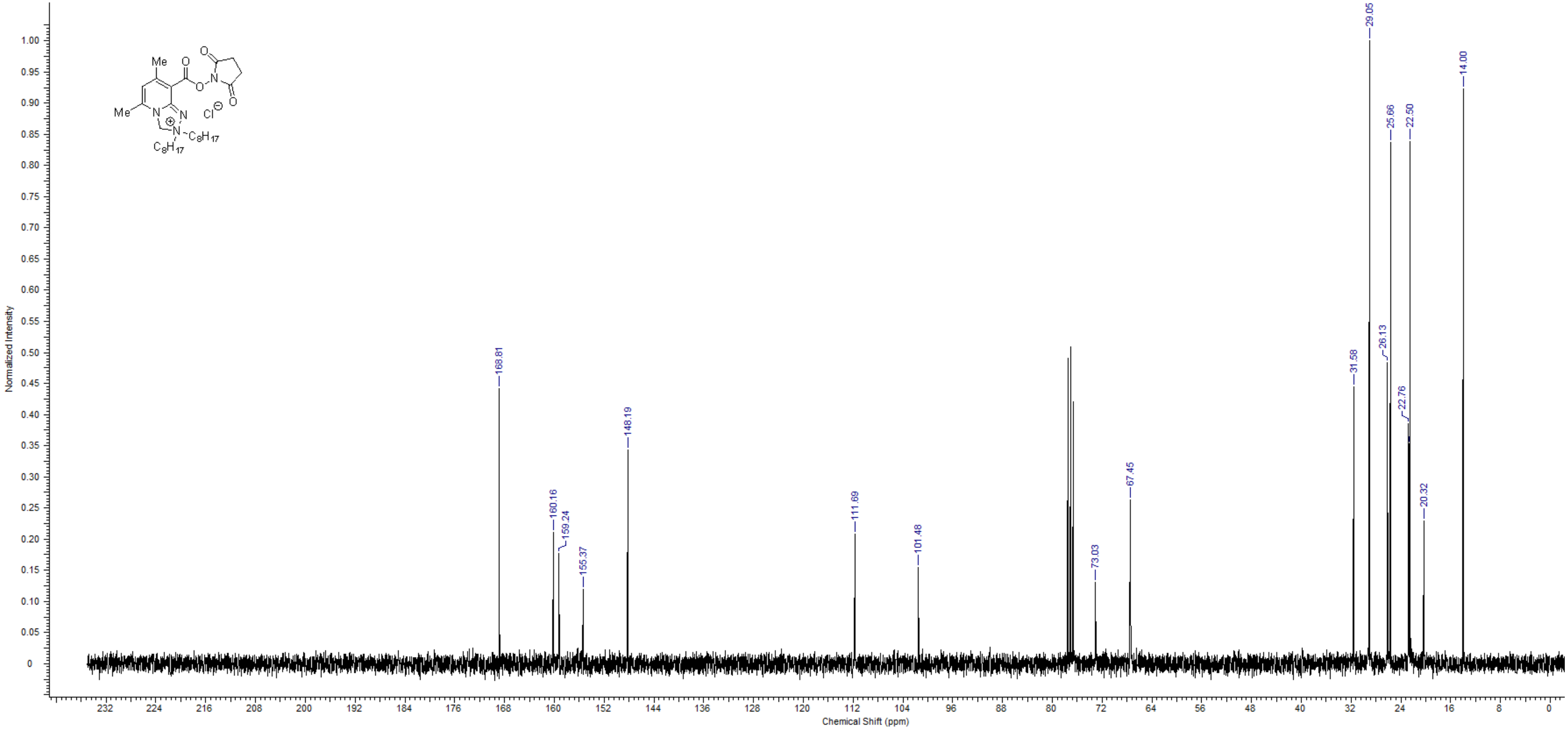

Figure S6. ^{13}C-NMR spectrum of **5b**

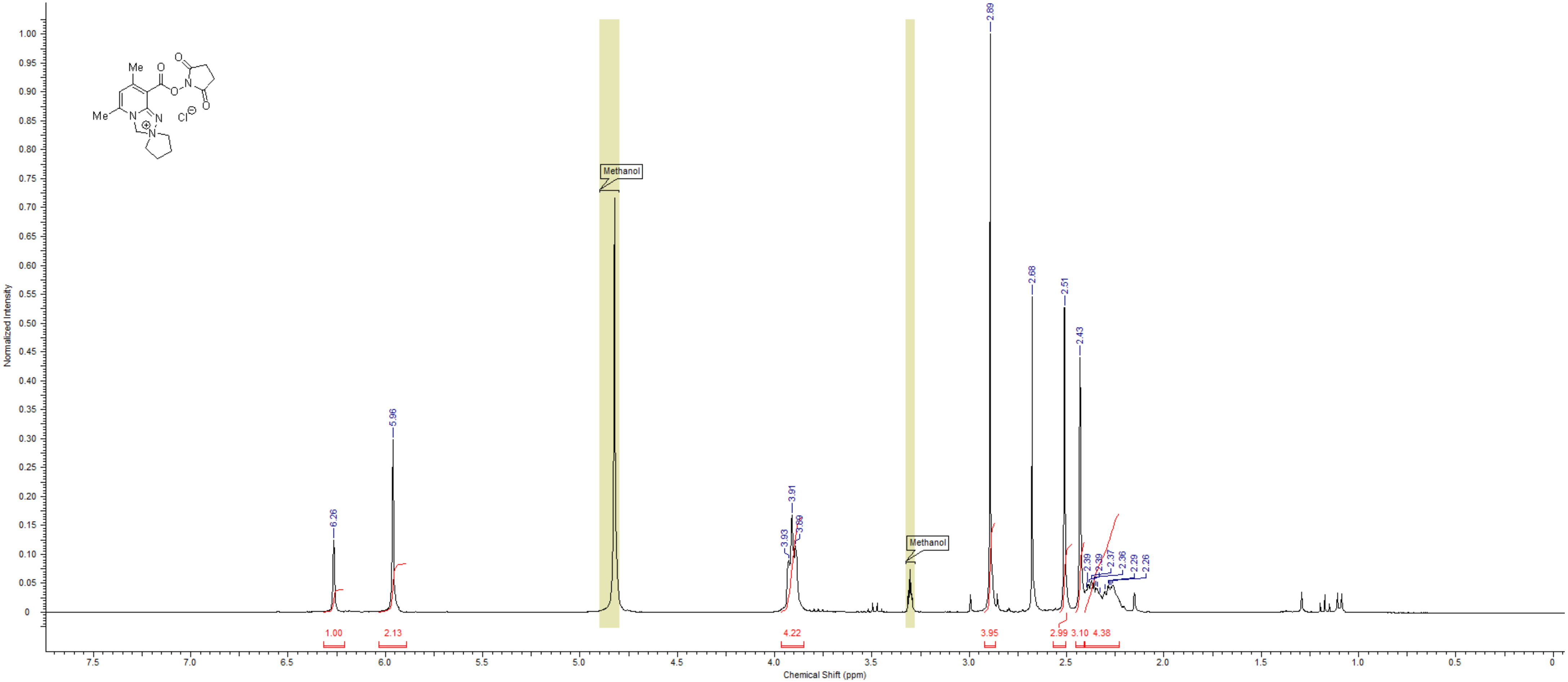

Figure S7. ^{1}H-NMR spectrum of **5c**

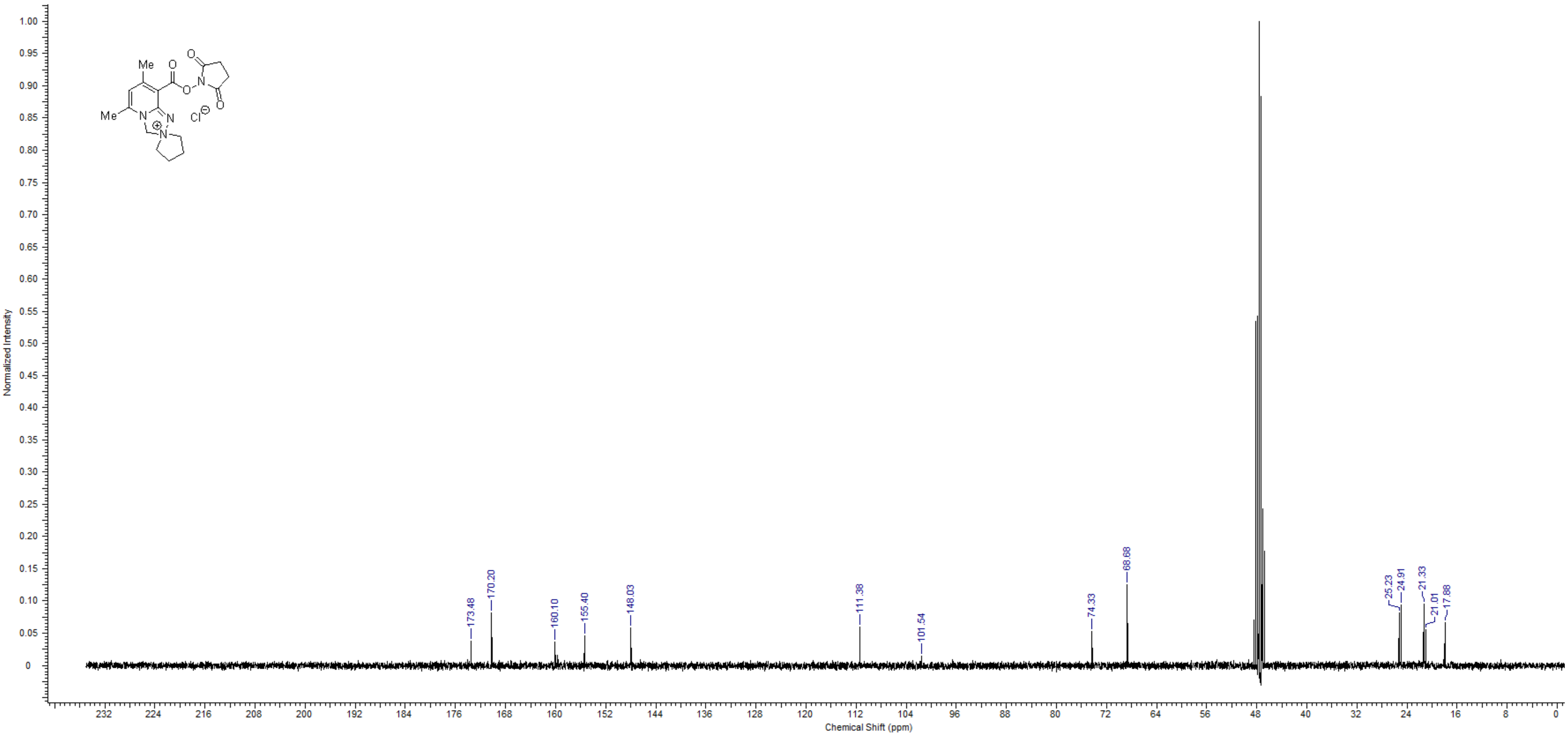

Figure S8. ^{13}C-NMR spectrum of **5c**

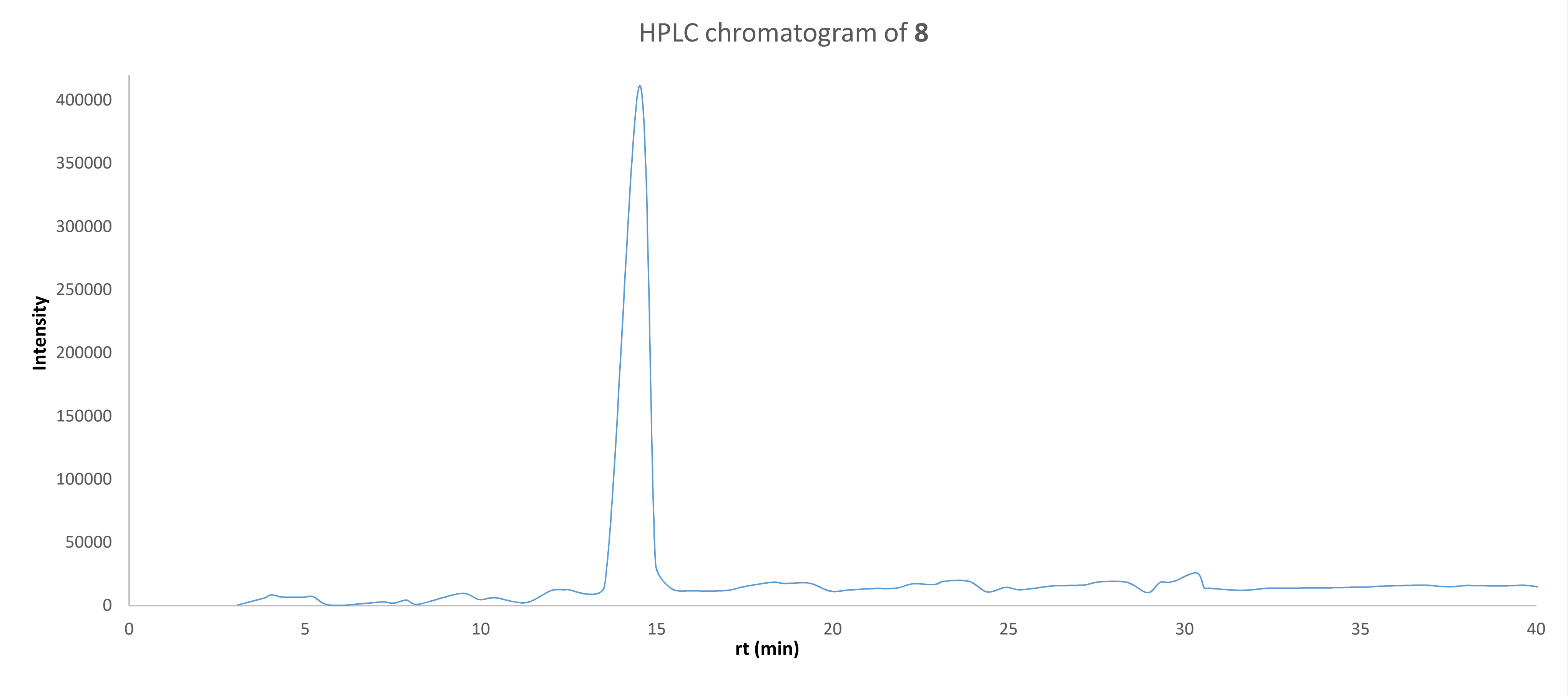

Figure S9. HPLC chromatogram of compound **8**

Video S1. Representative time lapse videos of PBS suspension of E. coli-pET30 bacteria labeled with *Safirinum* visualized by phase-contrast and fluorescence microscopy. The accelerated video corresponds to 2.5 sec. of phase contrast and 40 sec. of fluorescence original records. The focus was set to the dish bottom.
